# Supplementary material for: Ambulacrarian insulin-related peptides and their putative receptors suggest how insulin and similar peptides may have evolved from insulin-like growth factor
Source: PeerJ. 2021 Jul 14;9:e11799. doi: 10.7717/peerj.11799 (PMC8286064; doi:10.7717/peerj.11799)
Supplement: Supplemental Information 1 [file peerj-09-11799-s001.pdf]

Supplementary data for:

Ambulacrarian insulin-related peptides and their putative receptors suggest how insulin and similar peptides may have evolved from Insulin-like Growth Factor

by Jan A. Veenstra

Content

Spreadsheet 1 contains contains protein sequences for various insulin-like peptides and their putative precursors, as well as their genbank accession numbers or the terms Artemis or Trinity. Artemis indicates that the sequence in question was derived from a genome assembly, Trinity that the sequence was obtained using the Trinity program on a collection of transcriptome SRAs. In a few instances a combination of Trinity and Artemis was used to obtain a sequence.

Spreadsheet 2 contains the expression of some of these genes for a more limited number of species (there is little sense if the only transcriptome SRAs available for a species are from whole animals of unknown sex and unknown physiological status, or when then there are only very few such SRAs). The data contain the SRA identifier, the number of spots of each SRA and then for each protein of interest two numbers, one in blue, which is number of half spots that contains coding sequence of the protein of interest. The second number in black is the blue number multiplied by 1,000,000 and divided by the number of total spots, which yields a relative number.

The content of the remainder of this document is listed below:

The SRAs used in this study. 2

Figure S1. Sequences alignment of ambulacrarian IGFs. 6

Figure S2. Sequence similarity tree of ambulacrarian IGFs. 12

Figure S3. Sequence alignment of ambulacrarian GSSs. 13

Figure S4. Phylogenetic tree of ambulacrarian GSSs. 14

Figure S5. Sequence alignment of dilp7 orthologs. 15

Figure S6. Phylogenetic tree of ambulacrarian dilp7 orthologs. 16

Figure S7. Sequence alignment of ambulacrarian octinsulins. 17

Figure S8. Phylogenetic tree of ambulacrarian octinsulins. 19

Figure S9. Sequence allignment of ambulacrarian multinsulins. 20

Figure S10. Sequence similarity tree of ambulacrarian multinsulins. 24

Figure S11. Phylogenetic tree of bursicon and GPA2/GPB5 receptors. 25

Figure S12. Sequence alignment of various gonadulin receptors. 26

Figure S13. Sequence alignment of dilp7 receptors. 28

Figure S14. Sequence alignment GRL101 receptors. 32

SRAs used in the analysis:

Anneissia japonica
SRR9663012, SRR9663013, SRR9663014, SRR9663015, SRR9663016, SRR9663017, SRR9663018, SRR9663019, SRR9663020, SRR9663021, SRR9663022, SRR9663023, SRR9663024, SRR9663025, SRR9663026, SRR9663027, SRR9663028, SRR9663029, SRR9663030, SRR9663031, SRR9663032, SRR9663033, SRR9663034, SRR9663035, SRR9663036, SRR9663037, SRR9663038, SRR9663039, SRR9663040, SRR9663041, SRR9663042, SRR9897705, SRR9897706, SRR9897707, SRR9897708, SRR9897709, SRR9897710, SRR9897711, SRR9897712, SRR9897713, SRR9897714, SRR9897715, SRR9897716, SRR9897717, SRR9897718, SRR9897719, SRR9897720, SRR9897721 and SRR9897722.

Antedon mediterranea
SRR6650067.

Apostichopus japonicus
ERR1193930, ERR1193931, ERR1193932, SRR166825, SRR414926, SRR414927, SRR414929, SRR414930, SRR771602, SRR771603, SRR771604, SRR771605, SRR771606, SRR863579, SRR934650, SRR934651, SRR1002331, SRR1002332, SRR1002333, SRR1657913, SRR1657914, SRR1657915, SRR1657916, SRR1657917, SRR1657918, SRR1657919, SRR1657920, SRR1657921, SRR1657922, SRR1657923, SRR1657924, SRR1657925, SRR1663411, SRR1663428, SRR1663429, SRR1663431, SRR1663432, SRR1663433, SRR1663436, SRR1663437, SRR1663438, SRR1663439, SRR1663440, SRR2006096, SRR2006097, SRR2006098, SRR2006099, SRR2006100, SRR2006101, SRR2006102, SRR2006103, SRR2006104, SRR2010219, SRR2012319, SRR2012383, SRR2012397, SRR2012405, SRR2014140, SRR2014144, SRR2014145, SRR2014150, SRR2014151, SRR2014153, SRR2014166, SRR2034609, SRR2089767, SRR2128001, SRR2128002, SRR5083072, SRR5083073, SRR5083074, SRR5083075, SRR5083077, SRR5083078, SRR5083079, SRR5083080, SRR5083082, SRR5083083, SRR5083084, SRR5083085, SRR5083086, SRR5083087, SRR5083088, SRR5817185, SRR5817186, SRR5817187, SRR5817188, SRR5817189, SRR5817191, SRR5817192, SRR5817193, SRR5817194, SRR5817195, SRR5817196, SRR5817197, SRR5817198, SRR5817199, SRR5817200, SRR5817201, SRR5817202, SRR5817203, SRR5817204, SRR5817205, SRR5817287, SRR5817295, SRR5839517, SRR5839518, SRR5839536, SRR5839538, SRR5839540, SRR5839546, SRR5839650, SRR5839684, SRR5839694, SRR5839713, SRR5839720, SRR5839725, SRR5839728, SRR5839730, SRR5839731, SRR5839732, SRR5839733, SRR5839734, SRR5839798, SRR5839799, SRR5839800, SRR5839801, SRR5839814, SRR5839840, SRR5839889, SRR5839890, SRR5839891, SRR5839892, SRR5839893, SRR5839897, SRR5839899, SRR5839900, SRR5839901, SRR5839902, SRR5839903, SRR5839904, SRR5839949, SRR5839950, SRR5839951, SRR5839952, SRR5839953, SRR5839954, SRR5840021, SRR5840022, SRR5840023, SRR5840024, SRR5840025, SRR5840027, SRR6004440, SRR6004441, SRR6004442, SRR6004443, SRR6004444, SRR6004445, SRR6004446, SRR6004447, SRR6004448, SRR6075435, SRR6075436, SRR6075437, SRR6075438, SRR6378158, SRR6856564, SRR6856565, SRR6856566, SRR6856567, SRR6856568, SRR6856569, SRR8258074, SRR8258075, SRR8258076, SRR8258077, SRR8258078, SRR8258079, SRR8258080, SRR8258081, SRR8258082, SRR8279868, SRR8801559, SRR8801560, SRR8801561, SRR8801562, SRR8801563, SRR8801564, SRR8801565, SRR8801566, SRR8801567, SRR9670443, SRR9670444, SRR9670445, SRR9670446, SRR9670447, SRR9670448, SRR9670449, SRR9670450, SRR9670451, SRR9670452, SRR9670453, SRR9670454, SRR9670455, SRR9670456, SRR9670457, SRR9670458, SRR9670459, SRR9670460, SRR9670461, SRR9670462, SRR9670463, SRR9670464, SRR9670465, SRR9670466, SRR9670467, SRR9670468, SRR9670469, SRR9670470, SRR9670471, SRR9670472, SRR9670473, SRR9670474, SRR9670475, SRR9670476, SRR9670477, SRR9670478, SRR9670479, SRR9670480, SRR9670481, SRR9670482, SRR9670483, SRR9670484, SRR9670485, SRR9670486, SRR9670487, SRR9670488, SRR9670489, SRR9670490, SRR11793939, SRR11793942, SRR11793943, SRR11793944, SRR12505006, SRR12505011, SRR12505013, SRR12505016, SRR12505017, SRR12830751 and SRR12830752.

Asterias rubens

SRR3087891.

Florometra serratissima
SRR3097584.

Holothuria scabra
SRR9125585.

Holothuria arguiensis
SRR8889053, SRR8892978, SRR8893063, SRR8896868, SRR8896869, SRR8898365, SRR8898366, SRR8906597 and SRR8906598.

Lytechinus variegatus
SRR1139214, SRR1661401, SRR1661399, SRR1661397, SRR1661409, SRR1661406, SRR1661395, SRR1661363, SRR1661081, SRR1661075, SRR1661113, SRR1660833, SRR1661077, SRR1661079, SRR1660831, SRR1661112, SRR1661111, SRR1661090, SRR9673387, SRR9673388, SRR9673385, SRR9673386, SRR9673383, SRR9673384, SRR9673381, SRR9673382, SRR9673389, SRR9673390, SRR9673407, SRR9673408, SRR9673409, SRR9673410, SRR9673403, SRR9673404, SRR9673405, SRR9673406, SRR9673401, SRR9673402, SRR9673391, SRR9673392, SRR9673393, SRR9673394, SRR9673395, SRR9673396, SRR9673397, SRR9673398, SRR9673399, SRR9673400, SRR9673379, SRR9673380, SRR9673377, SRR9673378, SRR9673375 and SRR9673376.

Mesocentrotus nudus
SRR6438347, SRR6438348, SRR6438349, SRR6438350, SRR6438351 and SRR6438352.

Oligometra serripinna
SRR2845424.

Ophioderma brevispina
SRR10742213.

Patiria miniata
SRR1138704, SRR1138705, SRR1138706, SRR1138707, SRR1138708, SRR1139189, SRR1139190, SRR1139191, SRR1139193, SRR1139194, SRR1139195, SRR1139196, SRR1139197, SRR1139198, SRR1139199, SRR1139201, SRR1139214, SRR1139215, SRR1139455, SRR2454338, SRR5398132, SRR5398133, SRR5398134, SRR5398135, SRR5398136, SRR5398137, SRR5398138, SRR5398139, SRR5398140, SRR5398141, SRR5398142, SRR5398143, SRR5398144, SRR5398145, SRR5398146, SRR5398147, SRR5398148, SRR5398149, SRR5986254, SRR6054712, SRR8580044, SRR8580045, SRR8580046, SRR8580047, SRR8580048, SRR8580049, SRR8580050, SRR8580051, SRR8580052, SRR8580053, SRR8580054, SRR8580055, SRR8580056, SRR8580057, SRR8580058, SRR8580059, SRR8580060, SRR8580061, SRR8580062, SRR8580063, SRR8580064, SRR8580065, SRR8580066, SRR8926376, SRR8926377, SRR12983012, SRR12983013, SRR12983014, SRR12983015, SRR12983016, SRR12983017, SRR12983018, SRR12983019, SRR12983020, SRR12983021, SRR12983022, SRR12983023, SRR12983024, SRR12983025, SRR12983026, SRR12983027, SRR12983028, SRR12983029, SRR12983030, SRR12983031, SRR12983032, SRR12983033, SRR12983034, SRR12983035 and SRR12983036.

Patiria pectinifera
SRR1139200, SRR1141045, SRR5229423, SRR5229424, SRR5229425, SRR5229426, SRR5229427, SRR5229428, SRR8627925 and SRR8627926.

Pisaster ochraceus

SRR1139197, SRR2846074, SRR10982207, SRR10982208, SRR10982209, SRR10982210, SRR10982211, SRR10982212, SRR10982213, SRR10982214, SRR10982215, SRR10982216, SRR10982217, SRR10982218, SRR10982219, SRR10982220, SRR10982221, SRR10982222, SRR10982223, SRR10982224, SRR10982225, SRR10982226, SRR10982227, SRR10982228, SRR10982229, SRR10982230, SRR10982231, SRR10982232, SRR10982233, SRR10982234, SRR10982235, SRR10982236, SRR10982237, SRR10982238, SRR10982239, SRR10982240, SRR10982241, SRR10982242, SRR10982243, SRR10982244, SRR10982245, SRR10982246, SRR10982247, SRR10982248, SRR10982249, SRR10982250, SRR10982251, SRR10982252, SRR10982253, SRR10982254, SRR10982255, SRR10982256, SRR11293187 and SRR11293188.

Ptychodera flava
SRR1029584, SRR2033704, SRR2033705, SRR2033710, SRR2033711, SRR2033712, SRR2033714, SRR2033715, SRR2033716, SRR2033717, SRR2033718, SRR2033719, SRR2033720, SRR2033721, SRR2033722, SRR2033723, SRR2033724, SRR2033725, SRR2033726, SRR2033727, SRR2033728, SRR2033729, SRR2033730, SRR2033731, SRR2033732, SRR2033733, SRR2033734, SRR2033735, SRR2033736, SRR2033737, SRR2033738, SRR2033739, SRR2033740, SRR2033741, SRR2033742, SRR2033743, SRR2033744, SRR2033745, SRR2033746, SRR2079098, SRR2079099, SRR2079100, SRR2079101, SRR2079102, SRR2079103, SRR2079104, SRR2079105, SRR2079106, SRR2079107, SRR2079108, SRR2079109, SRR2079110, SRR2079111, SRR2079112, SRR2079113, SRR2079114, SRR2079115, SRR2079116, SRR2079117, SRR2079118, SRR2079119, SRR2079120, SRR2079121, SRR2079122, SRR2079123, SRR2079124, SRR2079125, SRR2079126, SRR2079127, SRR2079128, SRR2079129, SRR2079130, SRR2079131, SRR2079132, SRR2079133, SRR12736734, SRR12736735, SRR12736736, SRR12736737, SRR12736738, SRR12736739, SRR12736740, SRR12736741, SRR12736742, SRR12736743, SRR12736744, SRR12736745, SRR12736746, SRR12736747, SRR12736748, SRR12736749, SRR12736750, SRR12736751, SRR12736752, SRR12736753, SRR12736754, SRR12736755, SRR12736756, SRR12736757, SRR12736758, SRR12736759, SRR12736760, SRR12736761, SRR12736762, SRR12736763, SRR12736764, SRR12736765, SRR12736766, SRR12736767, SRR12736769, SRR12736770, SRR12736771, SRR12736772, SRR12736773, SRR12736774, SRR12736775, SRR12736776, SRR12736777, SRR12736778, SRR12736779, SRR12736780, SRR12736781, SRR12736782, SRR12736783 and SRR12736784.

Saccoglossus kovalewskii
SRR071700, SRR071701, SRR071702, SRR071703, SRR071704, SRR071705, SRR071706, SRR071707, SRR071708, SRR071709, SRR071710, SRR071711, SRR071712, SRR071713, SRR071714, SRR071715, SRR071716, SRR071717, SRR071718, SRR071719, SRR071720, SRR071721 and SRR071722.

Schizocardium californicum
SRR2921981 and SRR2922012.

Strongylocentrotus purpuratus
SRR409091, SRR409092, SRR409093, SRR409094, SRR409095, SRR409096, SRR505583, SRR505584, SRR505585, SRR505586, SRR505587, SRR505588, SRR505589, SRR531843, SRR531853, SRR531860, SRR531948, SRR531949, SRR531950, SRR531951, SRR531952, SRR531953, SRR531954, SRR531955, SRR531956, SRR531957, SRR531958, SRR531964, SRR531996, SRR532046, SRR532055, SRR532074, SRR532121, SRR532143, SRR532151, SRR533746, SRR1012313, SRR1012339, SRR1012340, SRR1012342, SRR1012401, SRR1012403, SRR1041572, SRR1041901, SRR1042009, SRR1043060, SRR1043069, SRR1139792, SRR1765910, SRR1765938, SRR1765978, SRR1765979, SRR1765980, SRR1765981, SRR1765982, SRR1765983, SRR1765984, SRR1765986, SRR1765988, SRR1765991, SRR2080791, SRR2080792, SRR2080793, SRR2080794, SRR2080795, SRR2080796, SRR2080797, SRR2080798, SRR2080799, SRR2080800, SRR2080801, SRR2080802, SRR2080803, SRR2080804, SRR2080805, SRR2080806, SRR2080807, SRR2080808, SRR2080809, SRR2080810, SRR2080811, SRR2080812, SRR2080813, SRR2080814, SRR2080815, SRR2080816, SRR2080817, SRR2080818, SRR2080819, SRR2080820, SRR2080821, SRR2080822, SRR2080823, SRR2080824, SRR2080825,

SRR2080826, SRR2080827, SRR2080828, SRR2080829, SRR2080830, SRR2080831, SRR2080832, SRR2080833, SRR2080834, SRR2080835, SRR2080836, SRR2080837, SRR2080838, SRR2080839, SRR2080840, SRR2080841, SRR2080842, SRR2080843, SRR2080844, SRR2080845, SRR2080846, SRR2080847, SRR2080848, SRR2080849, SRR2080850, SRR2080851, SRR2080852, SRR2080853, SRR2080854, SRR2080855, SRR2080856, SRR2080857, SRR2080858, SRR2080859, SRR2080860, SRR2080861, SRR2080862, SRR2080863, SRR2080864, SRR2080865, SRR2080866, SRR2080867, SRR2080868, SRR2080869, SRR2080870, SRR2080871, SRR2080872, SRR2080873, SRR2080874, SRR2080875, SRR2080876, SRR2080877, SRR2080878, SRR2080879, SRR2080880, SRR2080881, SRR2080882, SRR2080883, SRR2080884, SRR2080885, SRR2080886, SRR2080887, SRR2080888, SRR2080889, SRR2080890, SRR2080891, SRR2080892, SRR2080893, SRR2080894, SRR2080895, SRR2080896, SRR2080897, SRR2080898, SRR2080899, SRR2080900, SRR2080901, SRR2080902, SRR2080903, SRR2080904, SRR2080905, SRR2080906, SRR2080907, SRR2080908, SRR2080909, SRR2080910, SRR2080911, SRR2080912, SRR2080913, SRR2080914, SRR2080915, SRR2080916, SRR2080917, SRR2080918, SRR2080919, SRR2080920, SRR2080921, SRR2080922, SRR2080923, SRR2080924, SRR2080925, SRR2080926, SRR2080927, SRR2080928, SRR2080929, SRR2080930, SRR2080931, SRR2080932, SRR2080933, SRR2080934, SRR2080935, SRR2080936, SRR2080937, SRR2080938, SRR2080939, SRR2080940, SRR2080941, SRR2080942, SRR2080943, SRR2080944, SRR2080945, SRR2080946, SRR2080947, SRR2080948, SRR2080949, SRR2080950, SRR2080951, SRR2080952, SRR2080953, SRR2080954, SRR2080955, SRR3017856, SRR3017857, SRR3593587, SRR3593588, SRR3593589, SRR3593590, SRR3593591, SRR3593592, SRR3593593, SRR3593594, SRR3593595, SRR3593596, SRR3593597, SRR3593598, SRR3593599, SRR3593600, SRR3593601, SRR3593603, SRR3593604, SRR3593605, SRR3593607, SRR3593608, SRR3593610, SRR3593612, SRR3593613, SRR3593615, SRR3593616, SRR3593618, SRR3593619, SRR3593620, SRR8863027, SRR8863028, SRR8863029, SRR8863030, SRR8863031, SRR8863032, SRR8863033, SRR8863034, SRR8863035, SRR8863036, SRR8863037, SRR8863038, SRR8863039, SRR9595500, SRR9595501, SRR9595510, SRR9595511, SRR9595512, SRR9595513, SRR9595514, SRR9595515, SRR9595516, SRR9595517, SRR9595518, SRR9595519, SRR9693264, SRR9693265, SRR9693266, SRR10002625, SRR10002626, SRR10002627, SRR10002628, SRR10002629, SRR10002630, SRR10002631, SRR10002632, SRR10002633, SRR10002634, SRR10002635, SRR10002636, SRR10002637, SRR10002638, SRR10002639, SRR10002640, SRR10002641, SRR10002642, SRR10002643, SRR10002644, SRR10002645 and SRR10002646.

```
                      1
Anneissia             QPNHVRRYCG SELTSELERR CALKGGFNSP KKQESP---- ---------- ----------
Oligometra            -EPKYRYYCG TSLVQKLESL CAPR-GYNGP KPGTSV---- ---------- ----------
Antedon               -SPKYARFCG SDLASELQRR CRSQ-GYNGP QQNSPL---- ---------- ----------
Florometra            -KPKYARYCG ANLASELERR CRTR-GYNGP EQKTHL---- ---------- ----------
Lytechinus-1          ---SFPLLCG QELVNAVAFV CDGR-GYYGQ PS-------- ---------- ----------
Hemicentrotus-1       ----FPLLCG QELVKAVAAV CNDR-GYYGQ PS-------- ---------- ----------
Strongylocentrotus-1  ---SFPLLCG QELVKAVAAV CNDR-GYYGQ PS-------- ---------- ----------
Apostichopus-1        ---SGQRYCG EALLEAMAYV CGDR-GYFGV TSGIRGRS-- ---------- ----------
Holothuria-1          ADGTGQKYCG EALLEALAFI CGDR-GYYGM TSGIHGRS-- ---------- ----------
Asterias-1            ---SPVQLCG RELTETLRSI CGDR-GYYSP GQSFS----- ---------- ----------
Pisaster-1            ---APVQLCG KELTDALKSI CGER-GYYSP GQSFS----- ---------- ----------
Acanthaster-1         ---AVIQVCG NDLLDALKSV CGDR-GFYSP PPGYS----- ---------- ----------
Patiria-1             ---SPIRVCG NELLDALMSV CGDR-GLYSP PEGYS----- ---------- ----------
Ophiothrix-1          ----WQRLCG TFLVDVVSQV CGER-GTYAD DSTHDLRKRS LDNFVDRGKM YSAGISKTSD
Lytechinus-2          -----YRLCG RELADALAVV CKGR-GYYID DSEI------ ---------- ----------
Hemicentrotus-2       -----FRLCG RELADALAVV CKGR-GYYID DSEI------ ---------- ----------
Strongylocentrotus-2  ----SFRLCG RELADALAVV CKGR-GYYID DSEI------ ---------- ----------
Apostichopus-2        ----AQRYCG TNLADALRIV CADR-GYYTQ ----KGAF-- ---------- ----------
Holothuria-2          ----SQRYCG SNLADALKII CADR-GYYDQ HHTVHGAF-- ---------- ----------
Asterias-2            ----WPKICG EQLVETVSLV CSTR-GFYSH RDS------- ---------- ----------
Pisaster-2            ----WQKICG EQLVETVSLV CSTR-GFYSH RDS------- ---------- ----------
Acanthaster-2         ----WQRICG EQLVETVSIV CNTR-GFYSH R E------- ---------- ----------
Patiria-2             ----WQKICG EQLVETVSVV CSTR-GFYSH RVE------- ---------- ----------
Ophiothrix-2          -----SYLCG SQIVQMMAVV CEGR-GYYYT EGSTGQICN- ---------- --------IV
Amphiura              -MAMWGRLCG KPLADMVALV CDGR   YYT GQ-------- ---------- ----------
Ophioderma            -MGMWGRLCG AQLANMVSFV CEGR-GYYYG EE-------- ---------- ----------
Saccoglossus          ----WDKLCG RTLVDVLALI CNGR-GYNSG SPKK------ ---------- ----------
Ptychodera            ----WDRLCG RSLADMLALV CHGR-GYYTD VSRQQ----- ---------- ---------K
Schizocardium         ----WDKLCG RTLADMLSLV CHGR-GYYTD VSKHR-IR-- ---------- ----------

                     61
Anneissia             -------YLW NQEKRIEATS ---------- -TERAKEINI VYECCHSACT EAFLDSFCLS
Oligometra            -------NLF --GKKNK FT ---------- -TTASNKKDI VAECCDTPCS EEMLARFCLS
Antedon               -------LFQ --DKKPQISA ---------- -SIRSKEKDI IDACCLSRCT EAFLDSFCLS
Florometra            -------LFQ --DKKVQNIG ---------- -TIRSNERDI IEACCRSPCT EEFLDSYCLS
Lytechinus-1          --KRSAGVFE LESRAKTFLK SGISRGELGR SKRRVRTGLI VTECCHNRCS VSHLESYCNP
Hemicentrotus-1       --KRSAGIFE LETRAKTFLK SGISRGETRR SKRGSRTGLI VTECCLNRCS VSHLESYCNP
Strongylocentrotus-1  --KRSAGIFE LETRAKTFLK SGISRGETRR SKRGARTGLI VTECCLNRCS VSHLESYCNP
Apostichopus-1        ---VSRSPFL TEERANSFLT NE-------R TRKTRRTGRI VTECCDNPCS QRNLESYCNV
Holothuria-1          ---VSRSPFL SEERANSFLT ND-------G TRNRRGTGRI VTECCENYCT TSVLESYCNF
Asterias-1            ---------- ---------- ---------- RRAPT-HDGI ATRCCQSLCE SSILETYCNL
Pisaster-1            ---------- ---------- ---------- RRAPT-HDGI ATKCCQSLCE SSILETYCNL
Acanthaster-1         ---------- ---------- ---------- RRTPATQTGI ATRCCISYCE TSVLEKYCNP
Patiria-1             ---------- ---------- ---------- RRTPATQSGI ATRCCISHCD TALLETYCNK
Ophiothrix-1          TNNAESNPFR GESEAKSFLG L--------- SRQKRRTGRI VHECCNNICN YRIIESYCNP
Lytechinus-2          --AQKDSPIV PRHVASSFLG SSS-ASAHSR QRRRVRSGQI VNECCDNECS NNIMESYCNK
Hemicentrotus-2       --SQKDSPIV PHHVASSFLG SSS-ASAHSR QRRRVRTGQI VNECCDKECS NNIMESYCNR
Strongylocentrotus-2  --AQKDSPIV PHHVASSFLG SSS-ASAHSR QRRRVRTGQI VNECCDKECS NNIMESYCNR
Apostichopus-2        ------PPVI PRQRASSFLT TDS---ENHH PRRLRRGTGI VTECCEKACD REVLETYCNP
Holothuria-2          ------QAII PRETASSFLS TNT---ENHP RRIRRRTGQI VTECCINHCT RGILETYCNP
Asterias-2            --KRDVEVFQ NERAAKSFLG SRIG------ SRQRRRTGRI ATECCDRICS FDIVESYCNP
Pisaster-2            --KRDVELFQ NERAAKSFLG SRIG------ SRQRRRTGRI ATECCERVCS FDIVESYCNP
Acanthaster-2         --KREAELFQ DERTAKSFLG THIG------ SRQRRRTGRI ATECCEKVCS YDIVESYCNP
Patiria-2             --KREAELFQ DERTAKSFLS SHIG------ SRQRRRTGRI ATECCEKVCS FDIVESYCNP
Ophiothrix-2          YSFLTDPPLL TERQAKTFT- KSG------- ---RTRTGKI VTECCDNHCD MQIIESYCAP
Amphiura              ----TDSPLL SERQAKSFTS NGG------- ---RKRTGKI VTECCDKHCN LQIIESYCAP
Ophioderma            ----SDSPIL PERQAKAFT- NGG------- ---RPRTGKI VTECCDNHCN LQIIESYCAP
Saccoglossus          -KVKRESPFR SGMEANDFFG NIS-SKEKRR QRRRSGSGKI VDECCHQACD YTTLESYCAP
Ptychodera            SRRARETVFA TQEEANGFFG VG---S--GR TKRRRGSGLI VVECCDKICD YSTIESYCAP
Schizocardium         --LPRETIFQ TQEDAHKFFG ASV-FG--ER TQRRRGSGLI VAECCEKSCD YATIESYCAP
```

Figure S1. Sequences alignment of ambulacrarian IGFs. In order to illustrate the sequence similarities in the putative D- and E- domains of these molecules Seaview[1] was used.

[1] Gouy M, Guindon S, Gascuel O. 2010. SeaView version 4: A multiplatform graphical user interface for sequence alignment and phylogenetic tree building. Mol Biol Evol. 27:221-4. doi: 10.1093/molbev/msp259.

```
                      121
Anneissia             SNKDEDTTVE SDTV------ ---ETTTITG KKRKPTRKPK N-PLKS---- ----------
Oligometra            PTPKDNTIVE SETM------ ---ETTTIVN TKKQRTKKP- K-PTRK---- ----------
Antedon               SNPEDDITVE SETI------ ---ETTTIVG SKKQLKTKKP K-TTRK---- ----------
Florometra            STPVDDTTAE IETV------ ---ETTTIIG SKKPRTKKPK S-DRRK---- ----------
Lytechinus-1          LPQDAV---- ---H------ ---DPE---- -----VH--- -----IRLEK SAEEDAD---
Hemicentrotus-1       LQPDAV---- ---H------ ---DAE---- -----VH--- -----IRLEK SAEEDADET-
Strongylocentrotus-1  LPPDAV---- ---H------ ---DAE---- -----VH--- -----IRLEK SAEEDAD---
Apostichopus-1        ATTQTTEIPT ELTT------ ---EGT---- -----TTEPA ASPRRNSR-- NIEADG--TA
Holothuria-1          AT----ELPT ELST------ ---ERT---- -----TTEPS ASPRRNSR-- HAGADI--TP
Asterias-1            PAPPSQTQPS TAAP------ ---TTT---- -----TKMAP LTE--DRRTK DVVVDY---S
Pisaster-1            PAASQTQT-- QASS------ ---AAP---- -----TKMAP LTE--DRRTK EVVVDY---S
Acanthaster-1         PSTSQSQTA- ---------- ---AAP---- -----PRITT TPD--ERRAN EIVVDE---T
Patiria-1             PATPQSQTA- ---------- ---AAP---- -----QRITS TSD--DRRAN EIVVDP---P
Ophiothrix-1          WPTTTT---- ---T------ ---ATT---- -----TQSPE PLPNEQ--EG GYLTDEIQMK
Lytechinus-2          RTPEVPS--- ESAI------ ---SEI---- ---------- --P-----SG DRTEDSTVRT
Hemicentrotus-2       RTPEVPP--- ESAI------ ---SEN---- ---------- --P-----SE EITEDSTLRT
Strongylocentrotus-2  RTPEVPP--- ESAI------ ---SEN---- ---------- --P-----SE EITEDSTLRT
Apostichopus-2        HVTPTLALAS --LV------ ---TSLM-TK SPTPPSSEPS SSSSSSSSSR N--EDKFPMT
Holothuria-2          RVTLEPTRPA VTSL------ ---SPLN-PR SQEPSGVQPQ ESPEEVHQEN S--RDNYPMG
Asterias-2            WPVAIE---- ---S------ ---RDP---- -----PL-SP VAPGRVREDK SADVDYMYNP
Pisaster-2            WPV-VE---- ---S------ ---RDV---- -----SL-SP VAPGRVREDK SADVDNMY-P
Acanthaster-2         WPVVED---- ---R------ ---DDP---- -----ML-AP VAPGRVRQDK SADADLLLRP
Patiria-2             WPVVED---- ---R------ ---DDP---- -----MLLAP VAPGRVRQDK SADADELLLM
Ophiothrix-2          LQEGQVKFTS RNLD------ -FVNEAN-NV VEEPSVRVPQ QVE-----EN KIVEETVPYV
Amphiura              LPEGQTRTQL RHWFLHEKEK ESANEEN-NT VEELPPPPPP R-------SE ERDEEQVPDV
Ophioderma            LPEGQTRGPE RHEFLHEKER ETITDQN-NR VEPPPLV-AE E-------AI VEEEETVPVI
Saccoglossus          LPEGVVA--- DDSL------ ---KRF---- ---------- --L-----SQ SFGNDFKDTA
Ptychodera            WPKDIDP--- AKKI------ ---EGF---- ---------- --K-----EG TWEEEDY---
Schizocardium         WPKDMDP--- ALRF------ ---AGF---- ---------- --K-----YG SWEDEDY---

                      181
Anneissia             -RK------- ----KPKGSS ---------- ---------E INSE-----Q SA----SNTE
Oligometra            SGR------- ----KNKDDP ---------- ---------D -TSE-----E SA----SNKD
Antedon               SGK------- ---------- ---------- ---------- ---------- SE----KNRD
Florometra            DGR------- ----KSKGGS ---------- ---------D -MSD-----D SA----SNRD
Lytechinus-1          -----EGRPQ --DG----PS ---------- --QL-----D ---------- ----------
Hemicentrotus-1       -----QGRPQ --DG----PS ---------- --QL-----D ---------- ----------
Strongylocentrotus-1  -----EGRPQ --DG----PS ---------- --QL-----D ---------- ----------
Apostichopus-1        AAA--GGGSG QGNGRGKGKG ---------- RHGK-----G NRRE-----Q DQ------TV
Holothuria-1          DGE--TPR-Q SNNGRNRGNP ---------N RHSE-----D N--------P ED------NI
Asterias-1            DQL---ATEG SQMSRVDGVL -----THDT- --------VT NRSKT-TTES NE--GSYDNE
Pisaster-1            DQL---VIEG SQMSRVDGAL -----TQDI- --------VT NRSKSATTES NE--GSYDNE
Acanthaster-1         GQT---GNTN SQMLRGGNAM -----GAGS- --------RA NGTKAPPTEV VD--GRSDDD
Patiria-1             SGH---TGDT APRGESAKAP -----GPSG- --------AA NATKTPVAEV DE--GMSVDE
Ophiothrix-1          HIT--RGQE- ---D----NS ---------- --V------D LLSE-----G SD------LR
Lytechinus-2          NDQSTERRTD DTDD----AT NLEVPSPDAD APDA-----T ATSDVERPLP EE----TTAV
Hemicentrotus-2       DGDSTEIRTD NTNP----AT NLEVPSPEAN APDA-----T ATSDVEQSRS DN----TTAV
Strongylocentrotus-2  DGESTEIRTD -TNP----AT NLEVPSPDAN TPDA-----T ATSDVEQPRS DN----TTAV
Apostichopus-2        DNA--LGEDY -EGPTNEGPL TSGEPTPTEN RIS------N GPRG-----P SS----NASS
Holothuria-2          DNV--VREDY -EPPTNGGAL TSGQPTPTEN RIS------S GLRE-----H SN----T-SI
Asterias-2            DVV--DVEEA --------NS ---------- --VI-----Q ---R-----E ED------LI
Pisaster-2            NAA--DVEEA --------NS ---------- --VI-----Q ---R-----E ED------LI
Acanthaster-2         DIA--EISE- --DK----SS ---------- --LL-----R QAAE-----K DE------PI
Patiria-2             QEA--AAGEQ FAEE----NS ---------- --LL-----R ---Q-----E EE------LI
Ophiothrix-2          SQA--IVGET TSD------G FDWDSDVSET HNILNGGGDM YINDQPRDDP S-----ENDE
Amphiura              SQD--LTF-- --S------D HDWEDSIESE --TLNEAIVM L-ADAPLTEQ ------NTEE
Ophioderma            NQE--LVD-T LSD------Q IDLEDSIESE TNLLNDALLM L-SDQVRENS EQRIGTENEE
Saccoglossus          NEDKLEI-VT VVRP----SH ---------- --DE-----M DGTE---TRI ED----NEHV
Ptychodera            -HRKYHP-ES VEQP----NA NPEEPTPEPT TTDL-----D KSLE---SES RD----NVDI
Schizocardium         -YRKYHP-EE FTQP----DL TTWIASTDSA NHDE-----H FTTA---TPS E---------
```

Figure S1-continued.

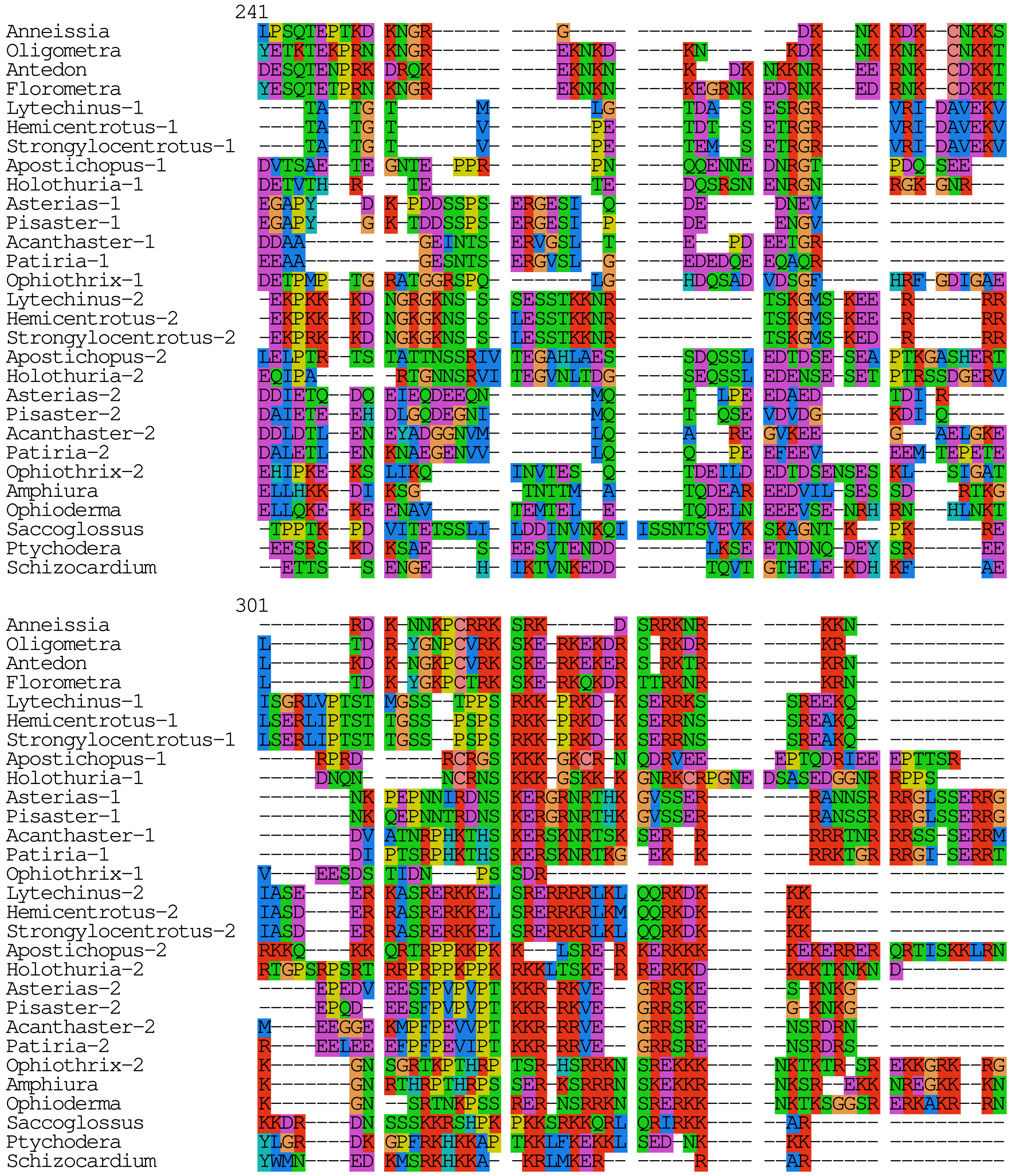

Figure S1-continued.

```
                         361
Anneissia                -----KKN-- ---------- ---------- -------KPT ---------- RRPKRWDSET
Oligometra               -----NKN-- ---------- ---------- -------KPT ---------- RRPKRWDSET
Antedon                  -----KKN-- ---------- ---------- -------KPT ---------- RRPKRWDSET
Florometra               -----KKN-- ---------- ---------- -------KPT ---------- RRPKRWDSET
Lytechinus-1             ---A-RREER RRNRE-RSSG GKSRSGRRKN K------D-- -ND-----RS SRAKRQGLTL
Hemicentrotus-1          ---A-RREER RRNRE-RSSG GRSRSGRRKD K------D-- -ND-----RA SRAKRHGLNM
Strongylocentrotus-1     ---A-RREER RRNRE-RSSG GRSRNGRRKD K------D-- -ND-----RA SRAKRHGLNL
Apostichopus-1           --EVSSTDGL -SRERGSGSG GGRNRG-N-- ---------- ---------- GKGKGKGSQR
Holothuria-1             --AATATEGA -DRSSGSRGG RGSNHQ-TGR G------S-- -DRGSSRSNG GKGKGNRNQD
Asterias-1               SSSSRREEKL RRRRQ----- --------RH RERELREQRK QSN--SKRKS KGDKKDHSV-
Pisaster-1               SSSSRREAKL RRRRQ----- --------RH RERELREQRK QSN--SKRKT KGDKKDHSA-
Acanthaster-1            LSSERKREDA TRKLR----- --------RK EQRLSRKQP- -HS--NKRKS KLE-KKGSE-
Patiria-1                LSIERKKDDA TRRRR----- ---------K EQRLLRKQQQ SNS--SKRRT KLE-KKGSE-
Ophiothrix-1             ---------- ---------- ---------- ---------- ---------- ----------
Lytechinus-2             ---KKRLESA ERNR------ -GTDHMGLSE DSTLLAREPL GID--VR--- ---KRFH---
Hemicentrotus-2          ---KKRLESA ERNR------ -GTDHMGLSE DSTLLAREPL GID--VR--- ---KRFH---
Strongylocentrotus-2     ---KKRLESA ERNR------ -GTDHMGLSE DSTLLAREPL GID--VR--- ---KRFH---
Apostichopus-2           EERAAGLNPT EQQEDGGGGG -GGGGGGGGG G------EPR NNSNSDQPRN NRGKVNESQR
Holothuria-2             --RGQRTNIK -KQKSGERGS ANPNPTRGGD R------EPT D----NEQSR NRGKVNESQR
Asterias-2               ---G-KSEGK NKKRSGSREG GRSSRRSRGK S------S-- -RS-----KK QRDGRERSKR
Pisaster-2               ---G-KSEGK NKRRSGSREG GRSSRRSRGK S------S-- -RS-----KK QRDGRERSKR
Acanthaster-2            ---G-KSEGK SKRKSGSREG GRSFRR-RGK N------S-- -RR-----KK GRDGRERSKR
Patiria-2                ---G-KSEGR SRKKSGSREG DRSSRR-RGK S------S-- -RR-----KK GRDGRERSKR
Ophiothrix-2             K-PKKNKNKH DRRRKGKRHG GD------RD DST----QPT ---------- ----------
Amphiura                 K-SKNK---K NRRRKN---R HG------ED DFT----QPT ---------- ----------
Ophioderma               K-PKHK---K NRRRKP---K HE------QD DYT----QPT ---------- ----------
Saccoglossus             ---GTRKTKL RHVKVKSKST PI-QQI---E TTTTM--KPF FDV---D-TD IIFYRFVNKT
Ptychodera               ---KSRAKSK NSTKVKPT-- -YVSSMTTSD EETLT--RPQ GDE--ARRET DATRKTEDSS
Schizocardium            ---KSRTKKG NNTKSKSILG KLVENM---- ---------- --E--TESPT NWQRDGTDER

                         421
Anneissia                TTLSDFLFSQ RFLQRAYDSD ID----VDEI TTEFGVVPEE DISSSLSEEE LSSNQVKSDD
Oligometra               TTLSEF---- --LQDIYNSD FADLSNIDKI KTGFDTIPEG DPSSSVSQEE SSSNEVIPDA
Antedon                  STLSDFLLSQ KLLQEIYDSD IDDRS-IDDI TTQFDALPED DLSSSLSQEE SSSNEVIQEA
Florometra               TTLSDFLLSQ RFLQGVYDSD IGDQSTLDEL APRFDAVQEG DTSYSLSQE- SSSNEVISEA
Lytechinus-1             WRN---MFSQ KALSN----- ---------- ---------- ---------- ----------
Hemicentrotus-1          WRN---MFSD KGFSN----- ---------- ---------- ---------- ----------
Strongylocentrotus-1     WRN---MFSD KFFSD----- ---------- ---------- ---------- ----------
Apostichopus-1           REQSEELTVE ------TTTG CASQDGQN-- ---CEESNAN AGGAS----- ----------
Holothuria-1             RNQTDEDAER NTSSE-NGHG SRQRGGGG-- ---EGEERRN NGGSSQRGGT SNSNTGTGNS
Asterias-1               -AATTPLAVQ ---------- ---------- ---E--RPLK NGGRNSTSGE HS-----SVN
Pisaster-1               -AVTTPLAVQ ---------- ---------- ---EPIHPLK NGGRNSTSGE LS-----SVN
Acanthaster-1            -SVTTPVAVQ ---------- ---------- ---TADHPFK HGAYNSTTGD LS-----LVN
Patiria-1                --VTTPVAIE ---------- ---------- ---TAEHPFK NGGYNSTSGD LS-----PVN
Ophiothrix-1             ---------- ---------- ---------- ---------- ---------- ----------
Lytechinus-2             ----HT---P RSSRE----- ---QAST--- ----ATHTSD DD-----PAT ------SRQE
Hemicentrotus-2          ----HT---P RSSRE----- ---QAST--- ----ATHALD DD-----SAT ------SRQE
Strongylocentrotus-2     ----HT---P RSSRE----- ---QAST--- ----ATHALD DD-----PAT ------SRQE
Apostichopus-2           HPN------- ---------- ---------- ---------- ---------- ----------
Holothuria-2             PAE------- ---------- ---------- ---------- ---------- ----------
Asterias-2               WEG---LDTS HPVKEPTARS V---LGR--- ---------- VDTRPFRNFL --YNRYTVDE
Pisaster-2               WEG---LDTS HPEKEPAARS V---FGG--- ---------- IDTRPFRNFL --YNRYTVDE
Acanthaster-2            WEA---LVTS HPPTKDIVDA LRSALRA--- ---------- AGRSPSRVA- ---GSFAPDE
Patiria-2                WEG---LVTS HPPTKEIVDA LRSALRA--- ---------- AGRTPSLFAP --ANNFLLDE
Ophiothrix-2             DEFITDIEVP HPRIQAVAGM PSSRSEPA-- ---NIDDPTD PGTRSERSFV ----------
Amphiura                 DELITDIEVM HPQARVVSRS EPVH------ ---AVDDPTD PGSRSERSFL ----------
Ophioderma               DQLITDIEVL HPQVQVVNAE SPSRSEAG-- ---NLDEAAD AGSRSERSFL ----------
Saccoglossus             WRVIEE---P EIMV------ ----GGK--- ----SESSRE SDIDSSI--- -------SHN
Ptychodera               WWMVDN---R EFEY------ ----EGR--- ----DRHNKS KTRK-GGNAT ------KVKK
Schizocardium            WWTVED---P HFQT------ ----GET--- ----DENSSN DKKTNGNNGT ------KWAR
```

Figure S1-continued.

```
                     481
Anneissia            QRTEHLPA-- ---------- ---------- ---------- -DI------- EVQ-SFDNIM
Oligometra           QRTDPFRP-- ---------- ---------- ---------- -PYKHILVDP IDM-DSYDNM
Antedon              QRTEQFRP-- ---------- ---------- ---------- -PLKPVPADT SED-SYNNNM
Florometra           QRTEQFRP-- ---------- ---------- ---------- -PFRPIPADT SEE-SYNNNM
Lytechinus-1         -------T-- ---------- ---------- -QALENQHIL DSLNGIAPSS TIIDTFETET
Hemicentrotus-1      -------I-- ---------- ---------- -PGIQNQLNL HQVNGRAPSS TTIDTFQMQT
Strongylocentrotus-1 -------I-- ---------- ---------- -PGLENQPNL HPVNGRAPSS TTIDTFQMKS
Apostichopus-1       -RRPGKTGKG K-GNGRRDD- A--DISSSER NTGDIEEEGT VPTSGNRPS- --S-------
Holothuria-1         QRTPGKPKKP QKGSSRRNQN Q--GSEEADE NTGAVSDEQS ESRNRVTPTQ TNS-------
Asterias-1           GTETDTA--- ---------- ---------G -AGSPEVKKD DLITTITAVL SDMI------
Pisaster-1           GTETDTA--- ---------- ---------- --GSPEVKKD DLITTITAVL SDMI------
Acanthaster-1        VTDSDTA--- ---------- ---------- --PSSDTKKD GFFTTITAVL RDVI------
Patiria-1            VTDPDSP--- ---------- ---------- --PSSDTKKD GFFTTITAVL RDVI------
Ophiothrix-1         ---------- ---------- ---------- ---------- ---------- ----------
Lytechinus-2         RRRTQARL-- ---SSRERKL HRTTTATARE ---ESQRDRR NVLQRL---- ----------
Hemicentrotus-2      RRRTQSRP-- ---SSRERKA HRTTTATARE --EELQRERR NVMQRL---- ----------
Strongylocentrotus-2 RRRTQSRP-- ---SSRERKT HRTTTATARE --EEMQRERR NVMQRL---- ----------
Apostichopus-2       -----VAQ-- ---------- ---------- ------EM-- NPLDKITKRI LAS-------
Holothuria-2         -----RQQ-- ---------- ---------- ------NN-- EEIDKLKKKI TFI-------
Asterias-2           KRDTERE--- ---------- ---------- -----SYRAV APLTGY-NSH RGG-------
Pisaster-2           KRDTDKE--- ---------- ---------- -----SYRAV APLTGY-NAY RGA-------
Acanthaster-2        QRSPLGTP-- ---------- ---------- -AGLQHRSGL DP-SGKPRPS YRV-------
Patiria-2            QRSPQATP-- ---------- ---------- -TGLQHRSAL DLSSGKAGRS YRV-------
Ophiothrix-2         ---------- ---------- ---------- ---------- ---TTIHARL VGF-------
Amphiura             ---------- ---------- ---------- ---------- ---TTITAKL LDV-------
Ophioderma           ---------- ---------- ---------- ---------- ---TTITAKL LDV-------
Saccoglossus         SRESHEP--- ----SREIDT T-------DC --DSV----- ---------- ----------
Ptychodera           ARHSQQTD-- ---DSRSSAE G-------GN --SEEMDVWH KPLTRPRGQP RRV-------
Schizocardium        KRRKDKPE-- ---PAVSRGR Q-------RR --SAVLGHRN SIFKRLRDRS WMV-------

                     541
Anneissia            S--------- -----RVNSK K---KTAAIK LKELTNQFSR LRQMQDATNA PRE-------
Oligometra           FM-----NSD DLSVQNVKPR L---EDPEEK IKKLTQIFSA --IVHDSTRT P---------
Antedon              YV-----STG NTR-NEQPPL D---DDPVEK LKELRHIFAE --LEGDSTSS S---------
Florometra           FM-----NAG NSR-KGEPSL K---DDPVAK LKKIKTYLHI SGLMQDSTSS S---------
Lytechinus-1         LLQHEKSLQD GAISHTKQPS INADEDRNAI FSDLMTKLR- -TLVLDFTSD R---------
Hemicentrotus-1      SSPTQQ---R NGGENFEQSS INQEADKKMR FSALMTKLR- -TMVLKFPRD R---------
Strongylocentrotus-1 SIPIQDSPEG NGKENFEQSS INQEADKKMR FSALMTKLR- -TMVLKFPKD R---------
Apostichopus-1       ---RES-SQS NGKGGGKKKL SKAEKKRQR- ---------- -KLLRKQEKA DRKAERKTGK
Holothuria-1         ---NESSSRG GRGKGGGNKL SKAEKKRQR- ---------- -KLLRQQEKK RRKQERQTKS
Asterias-1           ---------- ---------- ---------- ---------- -GFQPD-NGN R---------
Pisaster-1           ---------- ---------- ---------- ---------- -GFQPD-NGN R---------
Acanthaster-1        ---------- ---------- ---------- ---------- -GFQHADDGN R---------
Patiria-1            ---------- ---------- ---------- ---------- -GFQQADDGN R---------
Ophiothrix-1         ---------- ---------- ---------- ---------- ---------- ----------
Lytechinus-2         ---------- ---------- ---------- --------T- -GIFL----- ----------
Hemicentrotus-2      ---------- ---------- ---------- --------T- -GLFL*---- ----------
Strongylocentrotus-2 ---------- ---------- ---------- --------T- -GLFL----- ----------
Apostichopus-2       ---------- ---------- ---------- -------IH- -DIEMNREAD ID--------
Holothuria-2         ---------- ---------- ---------- ----REALR- -EVIIEPDSQ IG--------
Asterias-2           ---------- --SQ------ ----PDNHPT ----LAALY- -NLAVKLAKG LQH-------
Pisaster-2           ---------- --SQ------ ----PDNHPT ----LAALY- -HLAVRLAKG LQH-------
Acanthaster-2        ---------- --TQ------ ----KDNQST ----LEALY- -NLALRLSRP RRG*------
Patiria-2            ---------- --TQ------ ----KDNQST ----LEALY- -NLAVRLSRP RRG-------
Ophiothrix-2         ---------- ---------- ---------- --------L- -ESQQQNNNR RRR-------
Amphiura             ---------- ---------- ---------- --------I- -GLQQNSNHD RRR-------
Ophioderma           ---------- ---------- ---------- --------M- -GLQQNSNTN VR--------
Saccoglossus         ---------- ---------- ---------- ---------- ---------- ----------
Ptychodera           ---------- ---------- -E--VTA--- ---------- ---------- ----------
Schizocardium        ---------- ---------- -K--HEA--- ----RTHFS- -NLLLQAARR V---------
```

Figure S1-continued.

```
                          601
Anneissia                 ---------- ---------- ---------- ---------- ---------- ----------
Oligometra                ---------- ---------- ---------- ---------- ---------- ----------
Antedon                   ---------- ---------- ---------- ---------- ---------- ----------
Florometra                ---------- ---------- ---------- ---------- ---------- ----------
Lytechinus-1              ---------- ---------- ---------- ---------- ---------- ----------
Hemicentrotus-1           ---------- ---------- ---------- ---------- ---------- ----------
Strongylocentrotus-1      ---------- ---------- ---------- ---------- ---------- ----------
Apostichopus-1            SKKSKSKNKN RQTKGDRERR TTVRWNSNDE RSRRQVPRDS FSSVNGKYNS GHPYRRVYSM
Holothuria-1              SKK---GKKG SRTQGERERR TTVRWNDGEE RSRRHVPREQ FSPNNPRTTY HTDSR-TSLF
Asterias-1                ---------- ---------- ---------- ---------- ---------- ----------
Pisaster-1                ---------- ---------- ---------- ---------- ---------- ----------
Acanthaster-1             ---------- ---------- ---------- ---------- ---------- ----------
Patiria-1                 ---------- ---------- ---------- ---------- ---------- ----------
Ophiothrix-1              ---------- ---------- ---------- ---------- ---------- ----------
Lytechinus-2              ---------- ---------- ---------- ---------- ---------- ----------
Hemicentrotus-2           ---------- ---------- ---------- ---------- ---------- ----------
Strongylocentrotus-2      ---------- ---------- ---------- ---------- ---------- ----------
Apostichopus-2            ---------- ---------- ---------- ---------- ---------- ----------
Holothuria-2              ---------- ---------- ---------- ---------- ---------- ----------
Asterias-2                ---------- ---------- ---------- ---------- ---------- ----------
Pisaster-2                ---------- ---------- ---------- ---------- ---------- ----------
Acanthaster-2             ---------- ---------- ---------- ---------- ---------- ----------
Patiria-2                 ---------- ---------- ---------- ---------- ---------- ----------
Ophiothrix-2              ---------- ---------- ---------- ---------- ---------- ----------
Amphiura                  ---------- ---------- ---------- ---------- ---------- ----------
Ophioderma                ---------- ---------- ---------- ---------- ---------- ----------
Saccoglossus              ---------- ---------- ---------- ---------- ---------- ----------
Ptychodera                ---------- ---------- ---------- ---------- ---------- ----------
Schizocardium             ---------- ---------- ---------- ---------- ---------- ----------

                          661
Anneissia                 ---------- ---------- ---------- --------
Oligometra                ---------- ---------- ---------- --------
Antedon                   ---------- ---------- ---------- --------
Florometra                ---------- ---------- ---------- --------
Lytechinus-1              ---------- ---------- ---------- --------
Hemicentrotus-1           ---------- ---------- ---------- --------
Strongylocentrotus-1      ---------- ---------- ---------- --------
Apostichopus-1            DPRRTAA--H FRSKPKSVRQ IRRRPSSSSP RKVFIKAR
Holothuria-1              DQTRTSKTDQ HRGKHRKMSR NGKQLSRTRS KSSLLKPR
Asterias-1                ---------- ---------- ---------- --------
Pisaster-1                ---------- ---------- ---------- --------
Acanthaster-1             ---------- ---------- ---------- --------
Patiria-1                 ---------- ---------- ---------- --------
Ophiothrix-1              ---------- ---------- ---------- --------
Lytechinus-2              ---------- ---------- ---------- --------
Hemicentrotus-2           ---------- ---------- ---------- --------
Strongylocentrotus-2      ---------- ---------- ---------- --------
Apostichopus-2            ---------- ---------- ---------- --------
Holothuria-2              ---------- ---------- ---------- --------
Asterias-2                ---------- ---------- ---------- --------
Pisaster-2                ---------- ---------- ---------- --------
Acanthaster-2             ---------- ---------- ---------- --------
Patiria-2                 ---------- ---------- ---------- --------
Ophiothrix-2              ---------- ---------- ---------- --------
Amphiura                  ---------- ---------- ---------- --------
Ophioderma                ---------- ---------- ---------- --------
Saccoglossus              ---------- ---------- ---------- --------
Ptychodera                ---------- ---------- ---------- --------
Schizocardium             ---------- ---------- ---------- --------
```

Figure S1-continued.

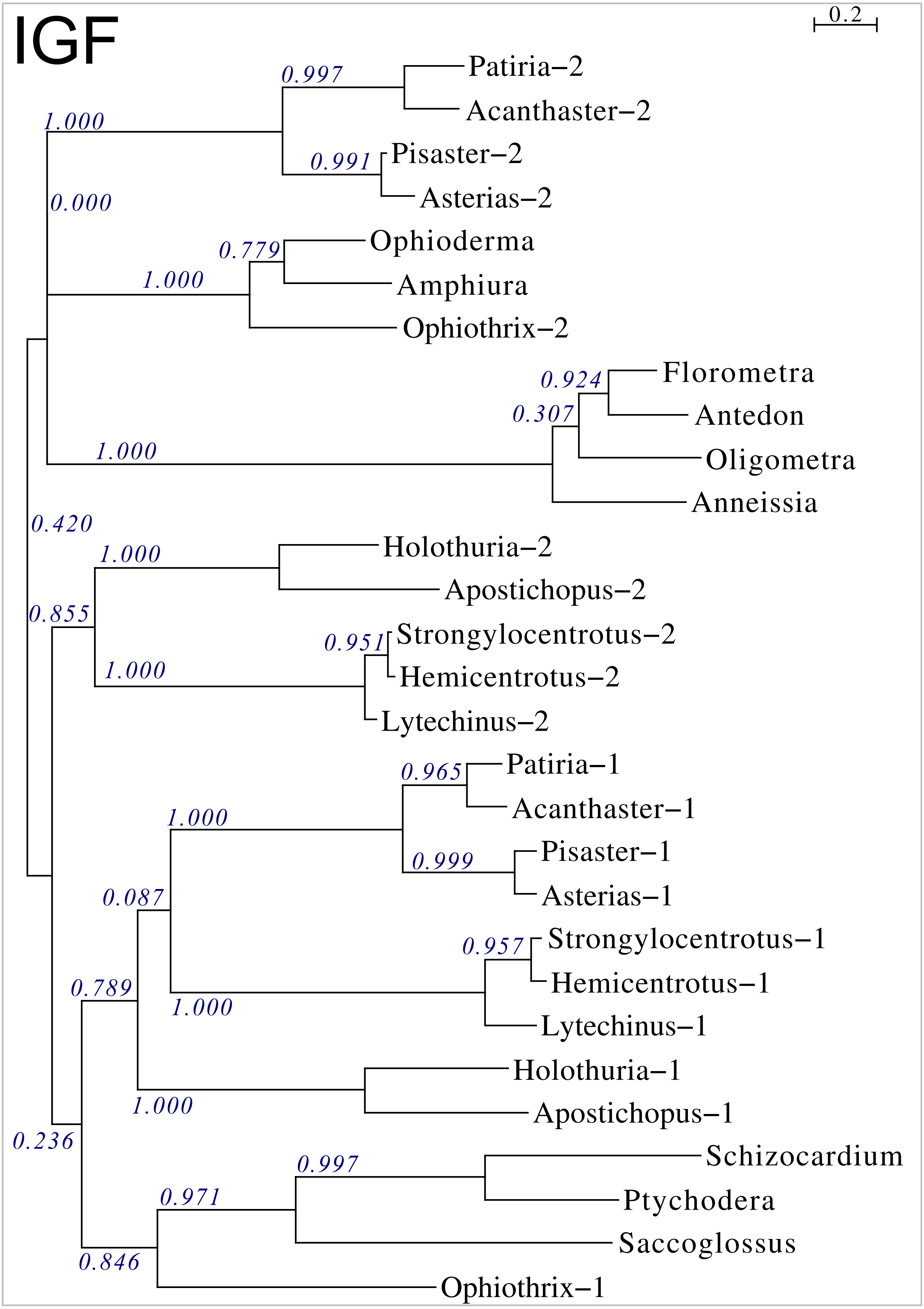

Figure S2. Sequence similarity tree of ambulacrarian IGFs.

```
Lytechinus            --QHLTRVRLCGLEFARAVYNHCSRASKRSDPGTISDIPLADRYLAR
Hemicentrotus         --QQGPRNRYCGLEFARAVFSQCSMANKRSDPGAVAESASAARYLAR
Strongylocentrotus    --QQGPRNRYCGLEFARAVFTQCSMANKRSDPGAVAESASAARYLAR
Apostichopus-1        -------IRLCGPDLSRAVYQICSHG-KRGYIPPTFNS--E------
Holothuria-1          -------VRLCGADLSRAVYRVCSHG-KRGYPMADL----E------
Apostichopus-2        ----WSHQRLCGPDLVHALSLVCGERG---YFGGSRLVER---D-VQ
Holothuria-2          -------HKLCGPALVDALAVVCRGRG---YYGGTVSRTK---REAS
Asterias-1            ------AEKYCDEDFHMAVYRTCTEH-KRS-GRSAFSL--N------
Pisaster-1            ------AEKYCDEDFHMAVYRTCAEH-KRS-GRSTYSL--N------
Acanthaster1          -------EKFCDNDFHLAVYQTCSTH-KRGDGEPVLSL--K------
Patiria-1             -------EKYCDDDFHMAVFRTCAVS-KRS--QPGMSL--S------
Asterias-2            RSDHASVKHFCGLEFSYAVVTACGEA-KRS-IRSAPFF---------
Pisaster-2            SNDHGRVKQYCGLEFSYAVVTACAEA-KRS-IRSAPFY---------
Acanthaster-2         ----DSSSKHCGSAFPQFVWTACSMA-KRS-NRSPRSL--D------
Patiria-2             ---TETTNRHCGAAFPDFVLAACSMA-KRS-IRSSPSL--H------
Ophiothrix-1          --DSARYQPLCGREFTRAVMEICATQVKRTEPLFQRFYNAN------
Ophiothrix-2          ----QDSYKSCGREFTRRVMEVCATHVKRTEHF--------------
Amphiura              -----DSAKYCGLAFSRAVMETCARQVKRTAPLWERLYTAS------
Ophioderma            -----DSATYCGVAFSMAVYETCSMQVKRTDPVRQRLYSAA------

Lytechinus            DTGYGQVQDTPFEWYDVAGQAEERLRP---------SL---SDIIFA
Hemicentrotus         DTGYEQAEDMPLEWYDIARQGAERLRP---------SL---TDIIFS
Strongylocentrotus    DTGYEQAEDMPLEWYDVARQGAERLRP---------SL---TDIIFS
Apostichopus-1        DDQLNQEFGTDL-----EEYLAETIKEYLKPNSLYDDVERELYPSLP
Holothuria-1          EEDFSQELDTEV-----DEFLAQALTGFLASRSFAADMESDRYYTLP
Apostichopus-2        DDGLDEEITTLV-------VGAERT-------SILECLK--AWSPFP
Holothuria-2          QDKLDV-------------------------------HA--GGSMTH
Asterias-1            D--F--------------------FRS-NSKRTAGSPRP--DDDFFL
Pisaster-1            D--LLSMD---------------SFRS-SPKRKAGSPLR--DDNSFL
Acanthaster1          D--VLTGS---------------RLRG-NIKRSFGSTLE--DEAFFA
Patiria-1             D--VLTMN---------------RFRGHNIKRSIDSTLE--DNAFFM
Asterias-2            D--MFPVF---------------KSPE-------RIPAD--FDDSSM
Pisaster-2            D--MFPVF---------------KSQE-------RIPAD--FDDSSM
Acanthaster-2         D--LLETF---------------KSAR-------HLDIS--Y--RTP
Patiria-2             D--LLQAF---------------KSDE-------YQANR--Y--TSP
Ophiothrix-1          ---LVKRSIDPAFWNNLLEANP--------------------DLMD-
Ophiothrix-2          ---MVKRSIDDEFWNDLMESGL--------------------GL---
Amphiura              ---RVKRFSDPEFWNAVLESDS--------------------DISM-
Ophioderma            ---RVRRFADPDFWEAVFNNDS--------------------NNVM-

Lytechinus            PRYRRSLFARPNTPMGRHCCVTGCTSQELASVC-------------
Hemicentrotus         SRFRRSIQNRRQLPMGQLCCVYGCTLVELASVC-------------
Strongylocentrotus    SRFRRSIHNRGQLPMGQLCCVYGCTLVELASVCT------------
Apostichopus-1        RG---FRRVTRTGGIARRCCSTGCSSSDIAKLC-------------
Holothuria-1          QR---FRRNA-RGGIARRCCASGCSSSDIAKLC-------------
Apostichopus-2        --------RRRTRGIVEECCFRRCTWENLESYCSKTTAYKKADNMI
Holothuria-2          --------VRFTRGIVEDCCFKGCTWSYLENYCLLS----------
Asterias-1            TM---QKRPETYVGMGSYCCLVGCTRDQLSQVC-------------
Pisaster-1            AL---QKRTETYVGMGSFCCLVGCTTEQLSQVC-------------
Acanthaster1          SR---LVKRSEYDGIASYCCIHGCTPSELAVVC-------------
Patiria-1             SG---LEKRSEYTGIASYCCLHGCTPSELSVVC-------------
Asterias-2            IH---VRKRQDYQGMATYCCTNGCTISQLTNSGIC-----------
Pisaster-2            IH---VRKRQDYQGMATYCCTNGCSISQLTNSGIC-----------
Acanthaster-2         IR---LSKRQDYDGMADYCCIIGCSTNELIASGIC-----------
Patiria-2             IH---LRKREEYMTIADYCCSVGCSPSDLVASGIC-----------
Ophiothrix-1          -----KRQSSAGVGMATHCCQSGCSQQEISMVC-------------
Ophiothrix-2          -----DKR--SETGMAEHCCQNGCTDQEISMVC-------------
Amphiura              -----DKRQPAGVGMATYCCNHGCTDTELSLVC-------------
Ophioderma            -----DKRQTTAMGMAQYCCSHGCTPQELSLVC-------------
```

Figure S3. Sequence alignment of ambulacrarian GSSs.

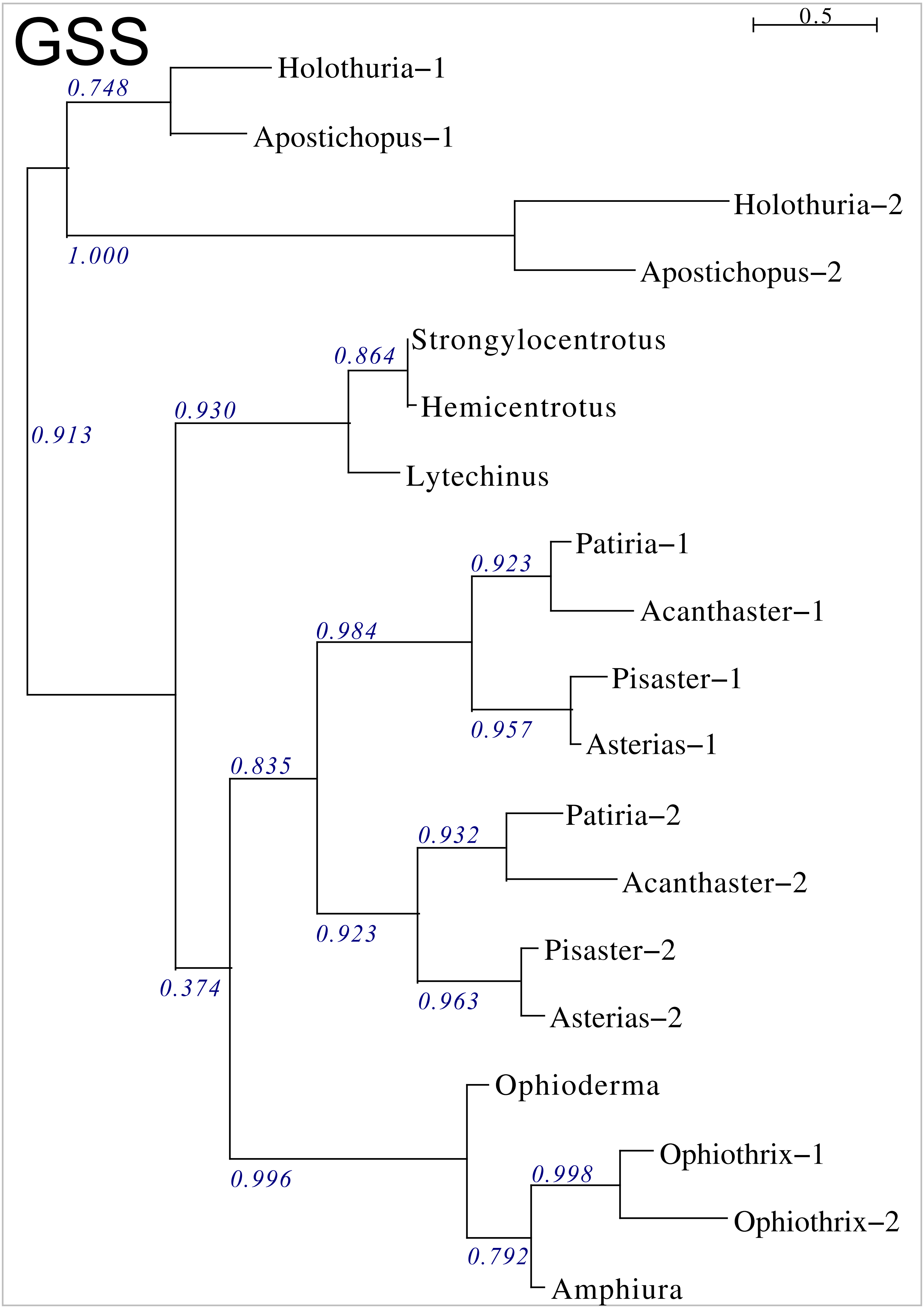

Figure S4. Phylogenetic tree of ambulacrarian GSSs.

```
Drosophila          ------LQHTEEGLEMLFRERSQSDWENVWHQETHS-R--C-RDKLVRQLYWA
Periplaneta         -------TKTENELEEMFKARSEEDWENAWHRERHT-R--C-QETLLRHLYWA
Stegodyphus         ------EVDVDPAWENIFKARSDEDWRSVWHTERHR-R--C-YHDLLVHMDWV
Oligometra          ---------------QDYSHRSRQEWARVWTVESMR-Q--C-HEDLREMVHIS
Anneissia           --------------LRDYSDRSHNDWARVWTVESMR-Q--C-HENLREMVHVS
Antedon             --------------LQDYSDRSHHDWARVWTVESMR-Q--C-HEDLREMVHIS
Florometra          --------------LQDYSDRSHYDWARVWTVESMR-Q--C-HEDLREMVHIS
Lytechinus          -DNLCNCQVIPQETMDEYELRTPEEWRENWLLDSLY-T--CVGPQLQRLGELA
Hemicentrotus       TEKFCNCMVIPELTMEDYEDRTPEEWRESWTMDTLR-T--CVGPQLQRVGELA
Strongylocentrotus  -EKFCNCMVLPELTMEDYEDRTPEEWRESWNMDTLR-T--CVGPQLQRVGELA
Apostichopus        -------------TLQELNSRTQPSWEQLWIVENV-PTVDCTV-DAVQLHIIA
Holothuria          -----DTTGLPETTIETLNAWTASEWVIAWASGSLVPVEGCTTLSVQQLFEYA
Asterias            --------AKPHLPVDQWRSRSKADWIRLWNTERHVNV--C-NEHLQPVFDAA
Pisaster            --------AKPHLPVDQWRSRSKADWIRLWNTERHVNV--C-NENLQPVFDAA
Acanthaster         ---------APHLPVEQWNSRSKADWVKLWNTERHVNT--C-NEALLPVWDVA
Patiria             --------AAPHLPVEQWKSRSKADWVKAWNTERHVDT--C-NEALLPVWDIA
Ophiothrix          -------------KITDYSSRTKADWQRLWLTESHQ-K--C-NEDLLPLWKIA
Amphiura            -------------RNTDYSSRTRADWQRLWVTESHQ-K--C-NEDLIPLWKIA
Ophioderma          -------------KTTDYSSRTTADWQRLWVTESHQ-K--C-NEDLIPLWKIA
Saccoglossus-1      LTVENEETITFSELNTMYGTRTLTDWQGKWTSETIH-A--C-GSNLYRISEYV
Ptychodera          ----AKDYDTVEDLILKYGTRTEDDWRNVWHTESHN-K--C-RNSLYSYVIFA
Schizocardium       ----ENAYETVESLKAKYSGRTFNDWRAAWNTETHN-K--C-RNSLYSYVIFA

Drosophila          CEKDIYRLTRRNKKRTGNDEAWIKKTTTEPDGSTWLHVNYANMFLRSRR----
Periplaneta         CEKDIYRLSRRNDYQQKENL-FLDE---GEPRYPFLSVVEARVFLRDRRRQR-
Stegodyphus         CQKDIYAVRRKKRST-----------------DPFLDDQKAHSFLGRRLKKSR
Oligometra          CRNDPRK-ITSKRN-------------------IFIPRSEATGFLSRFL----
Anneissia           CRNDPRK-ISSKRS-------------------IFIPRNEATGFLSRFL----
Antedon             CHNDPRK-ITSKRS-------------------IFIPRNEATGFLSRFL----
Florometra          CHNDPRK-ITSKRS-------------------IFIPRNEATGFLSRFL----
Lytechinus          CMNDPRKSVVVKRSN--------------SGREIFLPAKLAKAFLHYRHRHD-
Hemicentrotus       CINDPRKTIVVKRSN--------------SDRDLFLPAKLAKAFLQYRHRKD-
Strongylocentrotus  CINDPRKTIVVKRSN--------------SDRDLFLPAKLAKAFLHYRHRKD-
Apostichopus        CSNDVYKDHEGSRRRRF-------SDKRRRS--IFLNFSEANNFLA-KTKRT-
Holothuria          CQNDFYYDAEASTGL------GSGTGRRRRQSSIFLTHREASNFLLHRSKRFR
Asterias            CQNDVRK-IT-KRT-----------------GEEFVKEWAAKDFLIGS-----
Pisaster            CQYDVRK-IT-KRT-----------------GEEFVKEWTAKDFLIGS-----
Acanthaster         CQNDIRK-IT-KRM-----------------GREFLNEWTAKNFLAGS-----
Patiria             CQNDIRK-IS-KRT-----------------DREFVKEWNAKNFLAGS-----
Ophiothrix          CTYDIRK-IN-KRA------------------PEFVEDSEAKAFLIGP-----
Amphiura            CTYDIRK-IT-KRS------------------PEFIAEPDAKNFLLGP-----
Ophioderma          CTYDIRK-IN-KRS------------------PEFVAEPEAKNFLLGP-----
Saccoglossus-1      CYVDIHKSPDRKRTD-----------------DAFVDSAVAHDFLRGLMEKRT
Ptychodera          CMVDIYATGDKKRAE-----------------PPFVEHSIANSFLAGHRAEKR
Schizocardium       CMKDIHASGDRKRGQ-----------------FPFLDHEVADKFLSGSL-GKR

Drosophila          --SDGNTPSISNECCT-KAGCTWEEYAEY-CPSNKRRNHY-------------
Periplaneta         --RRGPGSSITEECCHNTAGCTWEEYAEY-CPANKRLRKFV------------
Stegodyphus         PHHTLVKRGIIDECCHGSAGCSWEEYAEY-CPANSRMRS--------------
Oligometra          --RTRRPSELHEDCCLDSRGCTWEEVAEIACINNRRRAHRPGSPIGR------
Anneissia           --RTRRPSELHEDCCLDSRGCTWEEVAEIACINNRRRMHRPGSPVGR------
Antedon             --RTRRPSELHEDCCLDSRGCTWEEVAEIACINNRRRMHRPGSPVGR------
Florometra          --RTRRPSELHEDCCLDSRGCTWEEVAEIACINNRRRIHRPGSPVGR------
Lytechinus          --SRRRRTGKDEECCLESNGCHLEELGEY-CTLHSRAYHVSGSQP--------
Hemicentrotus       --SRRRRVGKDEECCAEAQGCRWEELGEY-CTLHTRAYHQSGEQP--------
Strongylocentrotus  --SRRRRVGKDEECCAEAQGCRWEELGEY-CTLHTRAYHQSGEQP--------
Apostichopus        HSRVRRTTTFSTECCD--KLCIWEEVGEY-CWH-SRVYH--------------
Holothuria          RGRTRRSTTISAECCG-TQECKWEEIGEY-CVH-PREWHLP------------
Asterias            ----KRKRGLNEECCHEDTGCVWEEVAEY-CKKHGREKHKPGSTVAQGKQGR-
Pisaster            ----KRKRGLNEECCHEDTGCVWEEVAEY-CKQHGREKHKPGSPMVPGMQGR-
Acanthaster         ---KRRKRGLNEECCHEDLGCVWEEVAEY-CVMHGREKHEDGSPVRGKPGRRR
Patiria             ---RRRKRGLNEECCHEDLGCVWEEVAEY-CVMHGREKHEDGSPVRRRPGRRR
Ophiothrix          ---RRHKRGLNEECCHESKGCVWEEIGEY-CRMHSRASHVDGRIDSR------
Amphiura            ---IRHKRGLNEECCHEAKGCVWEEIGEY-CRMHSRASHVSGKVDAR------
Ophioderma          ---RRHKRGLNEECCHEAKGCVWEEIGEY-CRMHSRASHIDGRVDSR------
Saccoglossus-1      L-RRYRRTSATSECCADDGGCVWEELAEY-CTHQREVRTMDE-----------
Ptychodera          M-TRFRRASPTEECCSNAHGCNWEELAEY-CSHQRDDTRRR------------
Schizocardium       M-ARFRRANPSEECCSNRNGCTWEELAEY-CNHQRDDIDGR------------
```

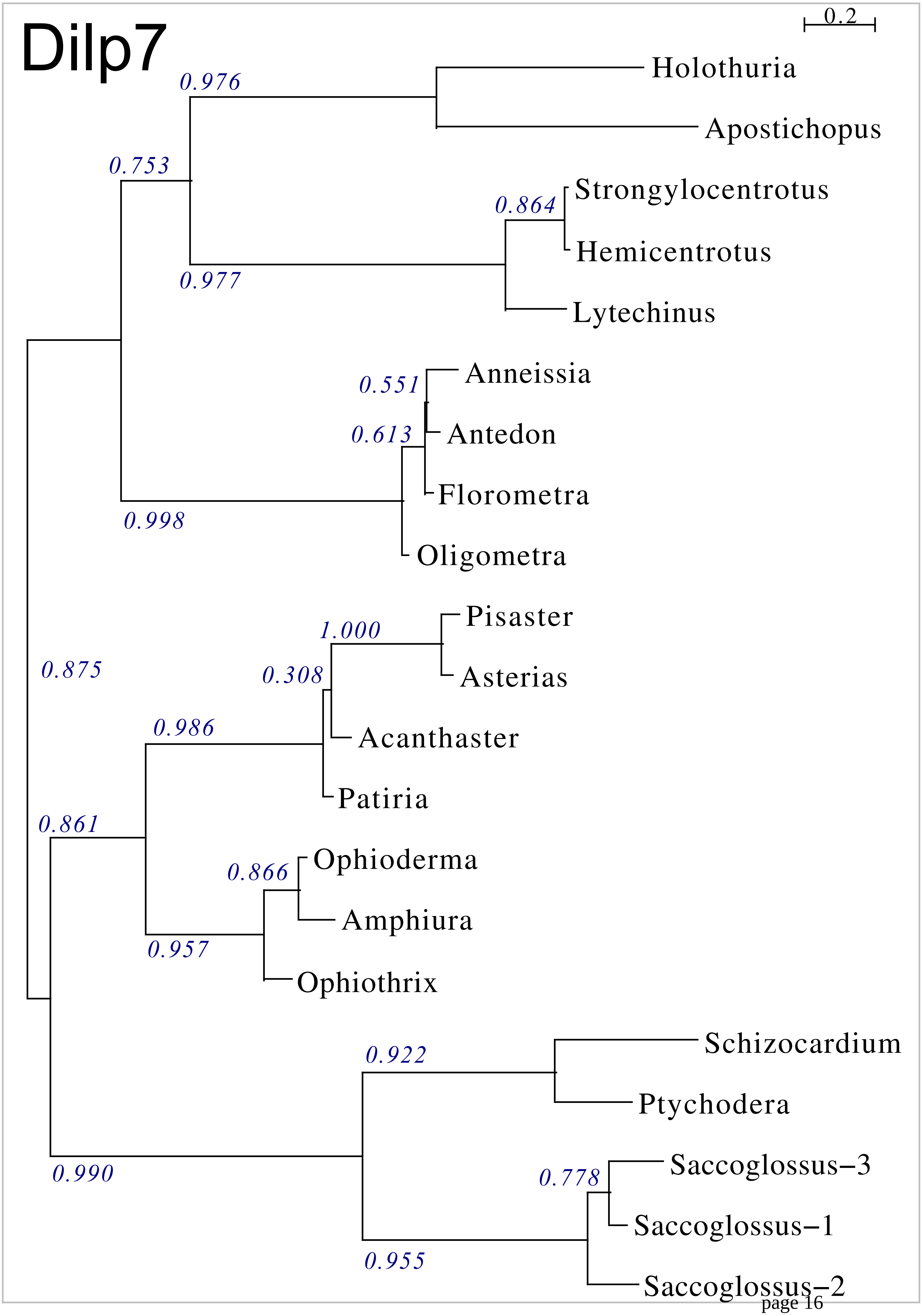

Figure S6. Phylogenetic tree of ambulacrarian dilp7 orthologs.

```
Anneissia            -------------------ARDWYC-GNAADTLKEFCQSCYASKRAHN--ALSLPSI
Oligometra           --------------------RDWYC-GNAATTLMDFCRSCYATKRAHT----SLPSS
Antedon              --------------------RDWYC-GSAANTLMNFCQSCYATKRSHS----SLPLL
Florometra           --------------------RDWYC-GNAATTLMDICQSCYATKRGYS----SLPSL
Lytechinus           --------------------QSWHC-GRAAQTIMSMCDSCYASYDKRS-------TS
Hemicentrotus        --------------------QSWHC-GRAAQTIMGMCNSCYASHDKRS-------IS
Strongylocentrotus   --------------------QSWHC-GRAAQTIMGMCNSCYASHDKRS-------IS
Apostichopus         ---------------------SWYC-GSAPETVRAICDGCYAGGIHTR--AFKRSSS
Holothuria           --------------------QSWHC-GSATETVRSVCNGCYAGGSHLSRNYLKRSHQ
Asterias             -------------------NNSWYC-SDVYSTLQSLCDSCYAGYAKRS-------DQ
Pisaster             -------------------NNSWYC-SDVYSTVQSLCDSCYAGYDKRS-------DQ
Acanthaster          --------------------DSWYC-SDVYSTVQSLCDSCYAGFDKRT-------NS
Patiria              ---------------------NWFC-SDVYSTMNSLCDGCYAGYDKRT-------NT
Amphiura             -----------------QRHERWYC-TPVFSMLQSMCGSCYAGIDKRS-------DN
Ophioderma           --------------------QRWYC-SPVFTMLQSMCGSCYAGIDKRS-------DD
Ophiothrix           --------------------NQWFC-SPVFTMLQSMCGSCYAGVDKRS-------DN
Saccoglossus-1       ------------------MSRNWHC-GRPVETMHEVCQGCYAGHVRPR-------NT
Ptychodera-1         ------------------LRREWHC-GRTVETMQGICRGCYAQPSERS-------T-
Schizocardium-1      ------------------FTKHWHC-GRIVDTMRAICDGCYASPTARD-------V-
Saccoglossus-2       RPSGSVD------------DVLTGC-KRKLLLVDQICAGCYAPPDIINNVNFDLTGK
Ptychodera-2         RPRNQGD------------D--VFC-SRTYSMVESVCDGCYATTQDSSPKSESAMNE
Schizocardium-2      RPNRPSE------------D--VRC-RKTIHMVLKLCNGCLAPIESEVENN--TISN
Saccoglossus-3       ---LPVDDTTTGVQVDVRDSRLWLC-GRLVEDLRALCRGCYAG---P----------
Ptychodera-3         -DLLPGNGDY---SNSAKGRRDLDC-THLVESMSLICRGCYATDQGV----------
Ptychodera-4         -----------------FLFNHRSCGTRLAETLSQICRGCYAHDDRP----------
Ptychodera-5         ----------------IRHRSGVSCGNRLRDSLASLCGGCYASDVR-----------
Ptychodera-6         -DLLPGNGDY---SNSANSRGNWHC-GRLVTTLSFICRGCYATGQ-V----------
Ptychodera-7         -DLLPGDGDY---SKSAKDRRDLDC-THLVDIMSFICRGCYATDQGV----------
Ptychodera-8         -DLLPGDGDY---SNSAKGRRDLDC-SLLIESMSFICRGCYATDQGV----------
Schizocardium-3      -------------QTSADRPRKWHCRKSVPEILSGVCRGCYAEPLQPP-------G-

Anneissia            KA-----KKDGMFLTKEGASGYLEAKRTRL--------FSS-LHLNHRQHETTNFVT
Oligometra           K------RTDGMFLTKERASGYLEAKRAKL--------FSSAVNLDRRQHAKTNFVT
Antedon              KS-----KSDEMFLTKERASGYLEAKRSRL--------FSS-VNLDRRQHGQTNFVT
Florometra           KS-----RTDGMFLTKERASGYLETKRSRL--------FSS-VNLDRRQHGQTNFVM
Lytechinus           KSSY---TPAKPFLHKRNAVHFLKSATREIENRPSIGDTAV-MSVERRSTGHRGFIH
Hemicentrotus        KSSY---TPAKPFLHKRNAVHFLKSTKREIESRPSIGDTAIEVAVERRSTGNRGFIH
Strongylocentrotus   KPSY---TPAKPFLHKRNAVHFLRTTKREIESRPSMGDTAIEVAVERRSTGNRGFIH
Apostichopus         DIIS---LYKDPFLKKSNALNFLLPRSHTP-------S-----SLIKRGIRRSGFIG
Holothuria           EKIP---LFKEPFLEKSYALNFLQAKSHAS-------DDPSPLMVRQRRSGRRGFID
Asterias             QTKT-----IDPFMDRKTAVDFFKRGTSR--------G-----------GARGGIID
Pisaster             QTKT-----IDPFMERKTAVDFFKRGASR--------G-----------GARRGIID
Acanthaster          ITRP---I-EEPFVERKNAVDFFKRGTAR--------G------------TRRGIVD
Patiria              ISRT---TDNEPFVERKTAVDFLKRGTAR--------G-----------STRRGIVD
Amphiura             VDRT-PQQSLDAFIQKEVAYSFIKRTSSG--------NS----FLRDSRSQHRGLID
Ophioderma           VDRT-QQQSLDAFIKKEVAHSFIKRTSAG--------SS----FLRNTR-QHRGLIE
Ophiothrix           SDTLSQKQSLDAFIQKEVAYSFIKRTSVG--------DT----FLRNARNTHHGLID
Saccoglossus-1       RS----VDGVQAFISRRDANMFTKGMSPD-------------V----KRAIDGGLIE
Ptychodera-1         -N----EAERQAFIGKEEASSFTKSVP---------------------TIVKRGLLE
Schizocardium-1      -G----SVKGLPFLKKHEASTFTRTRS---------------------SVQKRGIIE
Saccoglossus-2       --------------------------GPGY----------------EEEIVKNQIKE
Ptychodera-2         --------------------------SVQI----------------QDEDLREKIRD
Schizocardium-2      --------------------------ETPP----------------DQDVVTAKVRE
Saccoglossus-3       ------------DISKREASKFMQFNA-------------------HTIRQSRGIIE
Ptychodera-3         ------------TVNKRLASSFIPTTTTP-------------R-----NKIQRGIID
Ptychodera-4         ------------GISRREASSFLTTLRPQ-------------RAVARGSEERPNIVE
Ptychodera-5         -------------MTRRESSSFLQSK----------------------QRPLRGIVE
Ptychodera-6         ------------TVNKRLASSFIPTSTSP-------------R-----NKIQRGVID
Ptychodera-7         ------------TVDKRHASSFIPTTISP-------------S-----KKMQQDFIH
Ptychodera-8         ------------TVNKRLASSFIPTTTSP-------------R-----NKIQRGIID
Schizocardium-3      ---------KRDILNKQEASLFLRSP----------------------NGGDELIIN
```

Figure S7. Sequence alignment of ambulacrarian octinsulins.

```
Anneissia            ECCYNPCSSFEMIKYCCPTRQIELHNRNPNSSEDK----------------------
Oligometra           ECCYNPCSRFEMVKYCCTSRQIEFHESEGKK--------------------------
Antedon              ECCYNPCTRFEMIKYCCTSRQIEFNDTEGKK--------------------------
Florometra           ECCYNPCSRFEMIKYCCTSRQLEFNDTEGKK--------------------------
Lytechinus           ECCNKFCDPGEMILYCCEKRQIEWAHFHNLLKA------------------------
Hemicentrotus        ECCNKFCDPGEMVLYCCEKRQIEWAQFHNLLKA------------------------
Strongylocentrotus   ECCNKFCDPGEMVLYCCEKRQIEWAQFHNLLKA------------------------
Apostichopus         ECCEKNCEIREMVFYCCAEKQREYASFFPEIFRNRIRHT------------------
Holothuria           ECCRRSCDIFEMIFYCCAAQQQFYAEFFKLVKRS-----------------------
Asterias             ECCLKRCTTNELMLYCCEEKQREYFSFVGWLSRR-----------------------
Pisaster             ECCLKRCTTNEMMLYCCEEKQREYFSFVGWLARR-----------------------
Acanthaster          ECCHRQCAVSEMMLYCCEQKQREYYTFVGWLKRR-----------------------
Patiria              ECCHRPCSVNEMMLYCCEQKQREYYTFVGWLKRR-----------------------
Amphiura             ECCTSQCEAGEMILYCCQERQREWHSVRGFFNK------------------------
Ophioderma           ECCTQQCEIGEMVLYCCQERQREWHSMMGFFNK------------------------
Ophiothrix           ECCTQQCDTGEMILYCCQERQREWHIMMGLYNK------------------------
Saccoglossus-1       ECCYSQCSLTHMITYCCAEVQNEFQVFINILGNTDESSENDGDDGEESSSVHED---
Ptychodera-1         DCCYRRCNLQKMMTYCCAERQRELNNFFSLLNQKDNGST------------------
Schizocardium-1      HCCNHHCSFTELLIYCCEERSEEFYSFIGLLRMDDEDTDASLEKGNGDVEAEDNTDL
Saccoglossus-2       ACCKEYCPLPKIIEFCCDERQQEFHQFMSSFASTEE---------------------
Ptychodera-2         KCCNRRCTIHKMMQFCCEARRNEFHKFLALMGNTDN---------------------
Schizocardium-2      VCCDNYCSLDKIIEFCCEDLQQEFRQFMSFVSNSK----------------------
Saccoglossus-3       DCCYHTCPTERKIQYCCFEVQAQYRLFMESAI-------------------------
Ptychodera-3         ECCRNRCSVERKIQYCCDEIQKEFAFFFSLFGS------------------------
Ptychodera-4         ECCRRYCTLSRRIQYCCYEVQLEYQIYYESRNEE-----------------------
Ptychodera-5         ECCRSFCSLERRIQYCCYNVQARFAIFKEMSKEMSKERGM-----------------
Ptychodera-6         ECCRGTCSLERKIQYCCYEIQKEFALFKYGYRS------------------------
Ptychodera-7         ECCHTYCSFERKIQYCCNAVQQEFALFKSGYRS------------------------
Ptychodera-8         ECCHSRCSLQRKIQYCCDEIQEELALFLSLFGS------------------------
Schizocardium-3      ECCLRTCTVIEKIHYCCREKQIELYIIIQSAPWLVDNQR------------------

Anneissia            ------------------------------------
Oligometra           ------------------------------------
Antedon              ------------------------------------
Florometra           ------------------------------------
Lytechinus           ------------------------------------
Hemicentrotus        ------------------------------------
Strongylocentrotus   ------------------------------------
Apostichopus         ------------------------------------
Holothuria           ------------------------------------
Asterias             ------------------------------------
Pisaster             ------------------------------------
Acanthaster          ------------------------------------
Patiria              ------------------------------------
Amphiura             ------------------------------------
Ophioderma           ------------------------------------
Ophiothrix           ------------------------------------
Saccoglossus-1       ------------------------------------
Ptychodera-1         ------------------------------------
Schizocardium-1      DQGDDDNTQNNEGGSIDVVVERDVGDVLLKTDKSKP
Saccoglossus-2       ------------------------------------
Ptychodera-2         ------------------------------------
Schizocardium-2      ------------------------------------
Saccoglossus-3       ------------------------------------
Ptychodera-3         ------------------------------------
Ptychodera-4         ------------------------------------
Ptychodera-5         ------------------------------------
Ptychodera-6         ------------------------------------
Ptychodera-7         ------------------------------------
Ptychodera-8         ------------------------------------
Schizocardium-3      ------------------------------------
```

Figure S7-continued.

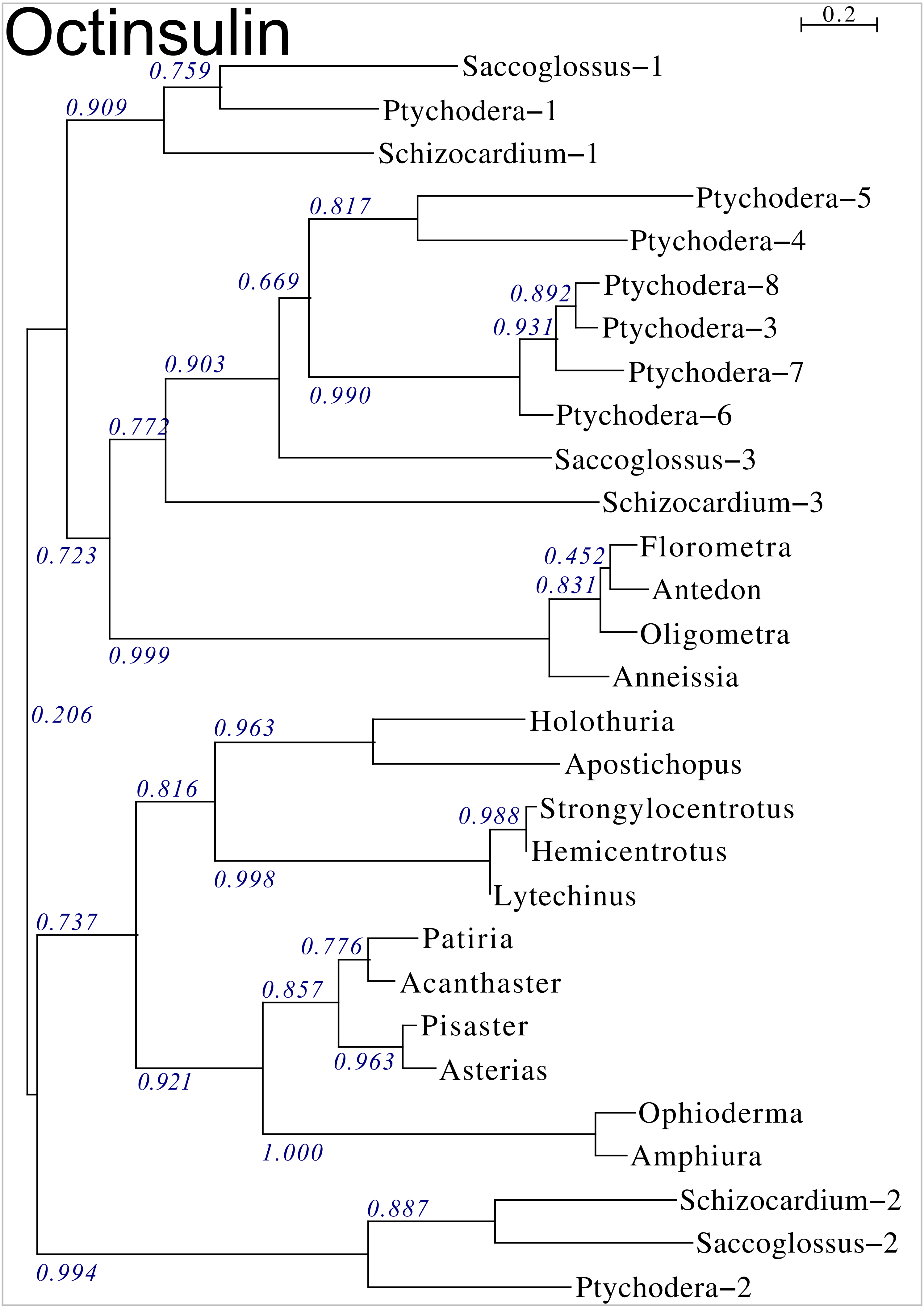

Figure S8. Phylogenetic tree of ambulacrarian octinsulins.

```
Amphiura-1     ---------------------------------------------------------
Amphiura-15    ------------------FD-----------LP------------------GREPGP
Amphiura-16    ----------------FLSDATSKKT-NEEALY------------------AQAMKR
Amphiura-2     ----------------YLTDAEH-------------------------------AER
Amphiura-3     ----------------YLTDAEH-------------------------------AER
Amphiura-7     YQDTDLRTLLDALEQSLRADSGDAPS-----------MKT-------------SKRA
Amphiura-6     IPDGYLRSLMDALQQIERADSGHAGV-DA------TAMKT-------------AERE
Amphiura-8     YQDTDLRTLLDELKQ--RADSRDAAA-----------IDT-------------NKRD
Amphiura-9     YQETDLRTLLEALKQ--REDSGDAPA-----------INT-------------NKKG
Amphiura-5     ------------------------------------------YP-----GALTRGTN
Amphiura-10    YPDTYLRTLLDALDQIVDSDATSKKT-NERAPDAYAMK-------------------
Amphiura-11    YPDTYLRTLLDALDQIVDSDATSKKT-NERAPDAYAMK-------------------
Amphiura-4     FPDNYLRTLLDALEKISDSDATGKRQ-MYADPDLARPPA------------------
Amphiura-14    FPDNYLRTLLDALEKIADSDATAKRG-LYDDEDLVRKPG------------------
Ophioderma-10  ---------------------DTETT-SSEEPDEWDEINP-TAIA--------ETKD
Ophioderma-3   -----------------------TKS-DNEEPDEWEVLHP-GE-------IREQSPD
Ophioderma-8   -----------------------TKS-ADEEPDEWEVLHP-GE-------PREPVSD
Amphiura-13    ------------------------KS-NSEEPDEHTT---------TTAPTTVGTDD
Amphiura-17    -----------------------TKS-IDEEPDELSVQGT-TTTGTTTAAVGRRDDD
Amphiura-18    -----------------------TKS-IDEEPDELSMQAT-T-----TVAARRRDDD
Ophioderma-1   ------------------DEIGAMKS-QNEEPDEHDSHIG-TT----QQ----PVIN
Ophioderma-2   -----------------------TKT-ANEEPDEWGLIPT-MP------PAPTDSLD
Ophioderma-4   -----------------------TNP-VNEEPDEGGLAP------------------
Ophioderma-7   -----------------------TTT-INEEPDEGGQAK------------------
Amphiura-12    -----------------------APNLFEDL-LRR--------DHVEQSGNITCM--
Acanthaster-5  ---------------------------VPRKPEEP--------SISAHCGMLLELRQ
Patiria-6      ---------------------------VPRKPGEP---------ISAHCGMLVELRQ
Asterias-4     -----------------------KLNLKPEETIDV--------TSSEHCGFVKEFRQ
Pisaster-2     -----------------------KLNMKPDEVLSV---------ESEHCSAVKEFRQ
Pisaster-5     ---------------------------------AQ--------QKSGHGPLLREVRQ
Asterias-1     -------------------------------------------ELRSQDGLMREVRQ
Pisaster-3     -------------------------------------------EPRPQDGLLREVRQ
Asterias-2     --------------------------------------------APNHNGLLREAKR
Pisaster-4     --------------------------------------------EPNHYGLLKETKR
Patiria-2      ----------------------DVPKPLA---DSE--------IDSNEDGEME----
Acanthaster-6  ----------------------DDPKPSESAAIAE--------DDSNKDGDIE----
Acanthaster-7  ----------------------DGPKLSES---------------TAIDSDIE----
Patiria-1      -----------------APPANDGIEVLTGGKIEE--------DDFEG----D-DFD
Acanthaster-2  ---------------------------------------------------LL-DLH
Acanthaster-1  ---------------------------------------------------HP-DAD
Acanthaster-8  ---------------------------------------------------HS-GAD
Acanthaster-9  ---------------------------------------------------HP-GAD
Patiria-5      ---------------------------------EP--------ADFNKDDKID-DLD
Patiria-8      -------------------------------------------------DKTD-DWH
Ophioderma-5   --------------NSLTRDADDAPVAMDDKPTSPRPIDIIKRSRHLRCAQFKDMAL
Ophioderma-6   ---------------------------------------AAVSPVHGSCVELK----
Ophioderma-9   ---------------------------------------AAFNSVPVSCADFQ----
Asterias-3     ------------------------------------------ELNVASCQQLADQVK
Pisaster-1     -----------------------------------------TELDEASCKQLADQVK
Acanthaster-3  ----------------------------------------DKTPSTASCEALAAQIS
Patiria-7      ----------------------------------------DKAPSAATCEALAATVK
Acanthaster-4  ---------------------SDTG--ADD---------DKVHIDDATCQALANQVK
Patiria-3      ---------------------DDAE--FKD---------GAIVIDDEVCEKLAAQVK
Patiria-4      ---------------------DDAE--FKD---------GAIVIDDEVCEKLAAQVK
```

Figure S9. Sequence allignment of ambulacrarian multinsulins.

```
Amphiura-1      ---------DWVGFRTICDPP-FTPSLYIEDFCGVT---VKR----------------
Amphiura-15     ASYV--ADNTWTGKTNICNYE-Q-QVVYHRELCGH----QKR----------------
Amphiura-16     YILN--PWSGWAGGKTECNTQ-TQESDF-DHACR------------------------
Amphiura-2      IDLA--KRKDW------CNNA---AHEF-SRLCN------------------------
Amphiura-3      IDLA--KRKDW------CNNA---AHEF-SRLCN------------------------
Amphiura-7      WEKE----FRSR---AECERQ-WK-LEL-DGHCISH----------------------
Amphiura-6      EGVK--RG----GVTPLCKSA-K--DRA-GKFCVYW----------------------
Amphiura-8      EGNE--KRFVSMSHSQACYSD-LYTYRK-PGICPTR----------------------
Amphiura-9      TDAR--D---------GCEAN-LN--VKVSQLC-------------------------
Amphiura-5      --DS--MNLRADFKLKLCGTE-LTLER--YYACGKR--DLK-----------------
Amphiura-10     ---R--GEYNWSGTKTLCGGT-LQTYK--GNICWGK--REAK----------------
Amphiura-11     ---R--GEYNWSGTKTLCGGT-LQTYK--GNICWGK--REAK----------------
Amphiura-4      --VF--DWQDWSGKKKICTAF-DQQQHF-NGVCQ------KRR---------------
Amphiura-14     --VF--DWEDWSGNKNICGRH-TQNQYY-NGVCQQYEGTGKRR---------------
Ophioderma-10   PVMM--RDKKWR-TSWFCGSD-IFRAK--KFICSPR--SYVRHIPY---------RKR
Ophioderma-3    IELD--HVKRWHSSVRICSS--KYRVQ--EAICDPR--NAKREVS-------------
Ophioderma-8    TELD--HVKRWHSSVRMCSS--MHRVK--ETICDPR--NAKREVS-------------
Amphiura-13     NAIL--MKRSWSGTKHLCGYQ-LNNER--SSVCGFK------RD--------------
Amphiura-17     DSST--LMKRWYGTVQLCGSH-LTKVK--SVICNPG--RPFKREE-------------
Amphiura-18     DSST--LMKRWNGYVQLCGSH-LTKVK--GVICNPG--APFKRDE-------------
Ophioderma-1    RSGT--MEKRWRGYVYICEPQ-LTRLK--NVICNPA--SVKRS---------------
Ophioderma-2    RSNM--LEKRWRGATKLCGST-LNTVQ--SVVCHPN--NYKRD---------------
Ophioderma-4    ------LGKRWSGSNKLCGEA-LSRAT--KNLCNPL--SAKRD---------------
Ophioderma-7    ------KGWSFTTGEKICGDD-LHTAT--TDVCFPD--SEKRD---------------
Amphiura-12     AYATMLEYEPNEEAYTWCDAN-LEHQR--EGICSCV--EYIKEHNKKRSLF-------
Acanthaster-5   MLDNPGLD-KRSPRNYWCGTT-LNNRK--SALCSCN--H------------------H
Patiria-6       MLDNPGLE-KRSPRNYWCGTA-LNNRK--STLCSCN--H------------------H
Asterias-4      LSDNPTLM-KRSPRNYWCSST-LERRK--EALCSCP--W------------------H
Pisaster-2      LADNPGVV-KRSPRNYWCNTA-LSQRK--TALCGCT--H------------------H
Pisaster-5      LFKGRNSQ-VRSETHYWCWEV-LEKNR--ESICNGP----------------PQPTQH
Asterias-1      LARNGRHF-RAAEDHIYCGVV-LEQNR--ESVCGVI----------------PGS---
Pisaster-3      LGRKPGHH-TRAEDHIYCGAV-LEQNR--EQVCGIV----------------PAS--R
Asterias-2      ILGRRGHQSRWDETHFYCDDA-LEQQR--EVICDPN--HTHAPVQPTPTGGPLPPTVS
Pisaster-4      VFRRRGFQPTNCEEAVEYCDA-LGQKR--EVLCQHN--HGTDAPRDSAVVG------A
Patiria-2       TKSGFSVA-WTGPQMWVCSSSTLAARK--HYVCHPP--GRHHHHKRE--------DE-
Acanthaster-6   AKGDFSVA-WSGPQMWVCRGR-LRRRR--RYACHPP--GRHHYKKRN--------DVQ
Acanthaster-7   AKGDFSVA-DNGPKLWVCQGN-VTNTL--KNVCTHT--GRPHSDTRD--------DVQ
Patiria-1       KNKNQYYS-RSGPRFRVCGTT-THSWS--SFVCHPS--GLI---------------HH
Acanthaster-2   AKEGRILV----RYPHYCNGI-LQIKY--NQLCHPQ--P-------------------
Acanthaster-1   AKHGGNLA----YRANYCGAT-LYEKV--RETCHAV--R-------------------
Acanthaster-8   TKEGGRVV----RYPHYCGAT-LQEKY--REICHAL--R-------------------
Acanthaster-9   TKEGGRVA----RYPHYCGAS-LQEKY--REICHAF--R-------------------
Patiria-5       EDDGQLET----RKPHYCGHR-LLRKY--AKICHHV--TLHRRPH-H---------LAA
Patiria-8       ADLG--QK----RSPHYCGRH-LGTKI--GQICGWH--L----------------EAW
Ophioderma-5    DHFRR-LVGKERVFAKYCSPT-PQTVM--DNYCQCD--VVPRSI-------------D
Ophioderma-6    ------KARELGAKHRFCGEE-LTYVR--NSYCSCR--VIPRDM-------------N
Ophioderma-9    ------SAQKRGNLHKLCGSE-LTAAV--DNYCQCK--VIPRDM-------------S
Asterias-3      EGKKRWD----GPSHKFCGET-LNEKR--YAYCTCG--LVPRKR-------------E
Pisaster-1      EGKKRWT----GSWHTFCGET-LNEKK--YAYCTCG--LVPRKR-------------E
Acanthaster-3   EGKKRWD----GPSHTFCGED-LNERR--NAYCNCQ--VVPRKR-------------E
Patiria-7       EGKKRWD----GPAHTFCGET-LTEKA--NAYCHCQ--VVPRKR-------------E
Acanthaster-4   ERGRRWPWSRTGPKHYFCQPT-LEKKK--EAYCNCK--VVPRKR-------------D
Patiria-3       EKS----ILGVGPKHWYCNPT-LERKK--EQYCTCS--VVPRER-------------D
Patiria-4       EKS----ILGVGPKHWYCNPT-LERKK--EQYCTCS--VVPRER-------------D
```

Figure S9-continued.

```
Amphiura-1      ------EYE-ATDPLGFLK-------MRGMVK---RLNP---NWRELMSEECCYESCT
Amphiura-15     ------EIE-AEDALGFLK-------MKLRGM---EKRG---GWQEYMNEECCIEWCT
Amphiura-16     -------LY-GRDALDFLK-------LRDVVK---RA--SFNDIKEWIHEECCLERCI
Amphiura-2      --------N-RRDALDFLN-------LRDVRN---KVHAFTNYIKEAVHEECCEESCI
Amphiura-3      --------N-RRDALDFLN-------LRDVRN---KLHAFTQYIKEGVYEECCDETCI
Amphiura-7      --PYRRDMP-DVDALAFLN-------KRG-------------KSLNGLYHECCDESCS
Amphiura-6      INRRDIEVS-MPDALGFL--------KTE-------------VQARDLNHECCVETCS
Amphiura-8      RSEDIQQLG-MQGALAFL--------KRE-------------MVKRDLFHECCVETCT
Amphiura-9      RREDIQQLD-TPDAFAFL--------KRE-------------LVQRDLYHECCVEQCD
Amphiura-5      --DALTFLE-DGA-----------------------------NRKRDLHEECCVETCS
Amphiura-10     --DALAFLE-TDDRQ----------------TDADP-LGIWALISTTLNEECCGEACY
Amphiura-11     --DALAFLE-TDDRQ----------------MDPLSTFISLALISTTLNEECCGEACY
Amphiura-4      --DIPQQLD-TPDALGFLQ-------AKRI-------AGFEKDVYESMTEECCLEGCT
Amphiura-14     --DIPQQLD-TPDALGFLQ-------AEK-----------RKDVFESINEECCAEQCS
Ophioderma-10   RSVGDATLI-KQDADMFLN-------RQKR-----------RKSSYSLYEECCGEFCS
Ophioderma-3    DNGVPGGVA-KETAKRFLK-------RQRR----------GEWGKGDLEEECCHEGCS
Ophioderma-8    DNGVPGGVA-KETAKRFLK-------RQRR----------GEWGKGDLEEECCHEGCS
Amphiura-13     AEDALDFLK-QDIV------------------------------KRSLTEECCHEGCS
Amphiura-17     SEAALAFLK-EERAKRFLQ-------ADQQLLHQQQLHQPSLRLWGGLTEECCNEHCS
Amphiura-18     GEAALAFLN-EERAKRFLQ-------PDQQ-----QLDQPNLRLWGGISEECCNEHCS
Ophioderma-1    DTAGLEFLT-EHQAKRFLM-------QNKR-----------IWGWGGLSEECCNEGCS
Ophioderma-2    --ETAALVG-EQQAKRFLR-------KR------------WWGGAGGLTEECCNEGCS
Ophioderma-4    --ETTAVME-ERRAKRFLI-----------------------EKRSSLNEECCNEGCA
Ophioderma-7    --A---IIQ-EKGAKRFLE-------NKRLW-------GWSWSTHGSLNEECCNEGCV
Amphiura-12     --KRMAEVT-EDVAKRFLESLLVLEVERAR------------RPKTSLQEECCNESCY
Acanthaster-5   SLLSDELIE-SKEANSFL--------KRGI------------EK-RSLTEECCHEGCY
Patiria-6       TFL-----K-SDEANNFL--------KRGV------------EK-RSLSEECCHEGCY
Asterias-4      TLRDDDFMEEKKDATNFL--------ERGV------------EK-RSLHEECCNEGCY
Pisaster-2      TLRDDDFMEEKKDATNFL--------ERGI------------EK-RSLSEECCHEGCY
Pisaster-5      YDSTTEFVG-AREAKGFL--------GKD---------------FTNLHEECCFEGCT
Asterias-1      --KRNLFVR-KEAASEFL--------ERG---------------VQGLAEECCGEGCT
Pisaster-3      LSKRNVFVG-KEAASDFL--------KRG---------------VQGLGEECCGEGCT
Asterias-2      PGHQDAFVG-KNEASRFL--------GRGV--------------VQGLIEECCFEGCH
Pisaster-4      IRSQDEFVK-RREASKFL--------GRGA--------------FQGLLEECCHEGCQ
Patiria-2       -DNSDAFLT-EDDANTFLA-------SPSK------------RKANIIHEECCHEGCF
Acanthaster-6   EESNPEFLD-EAEANFFLR-------R---------------HKRNHISEECCHEGCY
Acanthaster-7   EENNPEFLD-EADANFFLR-------Q---------------QNSKQISEECCHEGCY
Patiria-1       KRDNDEFLS-AGEANTFLM-------SEGV------------GK-RGMHEECCHEGCW
Acanthaster-2   EVFDQDFLD-SKEANTFLF-------KQRI------------GK-RSLIEECCFEGCT
Acanthaster-1   GVSNQEFLD-SKDASTFLF-------GRGI------------EK-RGLHHECCTEGCS
Acanthaster-8   GVSNQEFLD-SKDANTFLF-------GRGI------------GK-RNLNEECCSEGCD
Acanthaster-9   GVSNQEFLD-SKDANTFLF-------GRGI------------GK-RNLNEECCGEGCD
Patiria-5       NEAPSAFVE-SDEANSFLD-------NRGI------------SK-RGLHEECCHEGCS
Patiria-8       DDVHPAFVE-SDEANSFLA-------DRRI------------SK-RSLGEECCHEGCS
Ophioderma-5    --DKRAFVD-KSSAKSFLN--------HRA------------ST-RSLDEECCNEGCN
Ophioderma-6    P-------G-LEDAKAFLK-------MRSP------------ST-RSISEECCYETCY
Ophioderma-9    P-------G-LEDAKAFLK-------MRSP------------ST-RSISEECCYETCY
Asterias-3      -LELSEFLN-RGKANGFLS-------ARNV------------QK-RSLSEECCHEGCY
Pisaster-1      -LDLSEFLN-RGKANGFLS-------ARNL------------QK-RSLSEECCSEGCY
Acanthaster-3   -LDLSEFLP-SGKANAFLS-------GRRI------------TK-RSLSEECCHEGCY
Patiria-7       -LELSEFLT-PVKANSFLS-------GRNI------------AK-RSLTEECCHEGCY
Acanthaster-4   DGDQSEFLK-PEKAGSFLE--------SSI------------GK-RDFNHECCKEGCY
Patiria-3       -ADRAEFLE-PEEAGGFLN--------R--------------SK-RDLNHECCDEGCV
Patiria-4       -ADRAEFLE-PEEAGGFLN--------R--------------SK-RDLNHECCDEGCV
```

Figure S9-continued.

```
Amphiura-1       SEEIKELC-----------------
Amphiura-15      MEEVKELDC----------------
Amphiura-16      VEEVVELC-----------------
Amphiura-2       NEEVLEFC-----------------
Amphiura-3       DEEVLEFCP----------------
Amphiura-7       MEEFGEHCTQRPESQEGFNPFKLGK
Amphiura-6       DEEVKEHCVMTK-------------
Amphiura-8       TEEIQETC-----------------
Amphiura-9       AEEIEETC-----------------
Amphiura-5       MNEIEEDC-----------------
Amphiura-10      REEIAETC-----------------
Amphiura-11      REEIAETC-----------------
Amphiura-4       KGEVQELCP----------------
Amphiura-14      KHEIQELC-----------------
Ophioderma-10    MEELDETC-----------------
Ophioderma-3     MEEVNETC-----------------
Ophioderma-8     MEEVNETC-----------------
Amphiura-13      WEEIKETC-----------------
Amphiura-17      VEEINEVC-----------------
Amphiura-18      LEEVNEYC-----------------
Ophioderma-1     VEEIDEVC-----------------
Ophioderma-2     NEEVNESC-----------------
Ophioderma-4     SEEISEHC-----------------
Ophioderma-7     TEEIREHC-----------------
Amphiura-12      VQEVYDHCDKHFDGSI-YDLFRS--
Acanthaster-5    YEEVYELC-----------------
Patiria-6        YEEVYELC-----------------
Asterias-4       YEEVYELC-----------------
Pisaster-2       YEEVYEVC-----------------
Pisaster-5       VEEVNESC-----------------
Asterias-1       IEEISESC-----------------
Pisaster-3       VEEISESC-----------------
Asterias-2       SEEIHEHCP----------------
Pisaster-4       AEEIHEHCPKVY-------------
Patiria-2        WEEILEYC-----------------
Acanthaster-6    WEEILEYC-----------------
Acanthaster-7    WEEVLEYC-----------------
Patiria-1        WEEMLEHC-----------------
Acanthaster-2    NEEIYEVC-----------------
Acanthaster-1    IEEIYESC-----------------
Acanthaster-8    DEEIYET------------------
Acanthaster-9    DEEIYEIC-----------------
Patiria-5        NREVRKHC-----------------
Patiria-8        NEEVREHC-----------------
Ophioderma-5     LEEIVELSKTMCSSS----------
Ophioderma-6     YEEIEEVC-----------------
Ophioderma-9     YEEIEEVC-----------------
Asterias-3       WEEIEEVC-----------------
Pisaster-1       WEEIEEVC-----------------
Acanthaster-3    WEEIEEVC-----------------
Patiria-7        WEEIEEVC-----------------
Acanthaster-4    WEEVEEQCH----------------
Patiria-3        WEEVEEHCH----------------
Patiria-4        WEEVEEHCH----------------
```

Figure S9-continued.

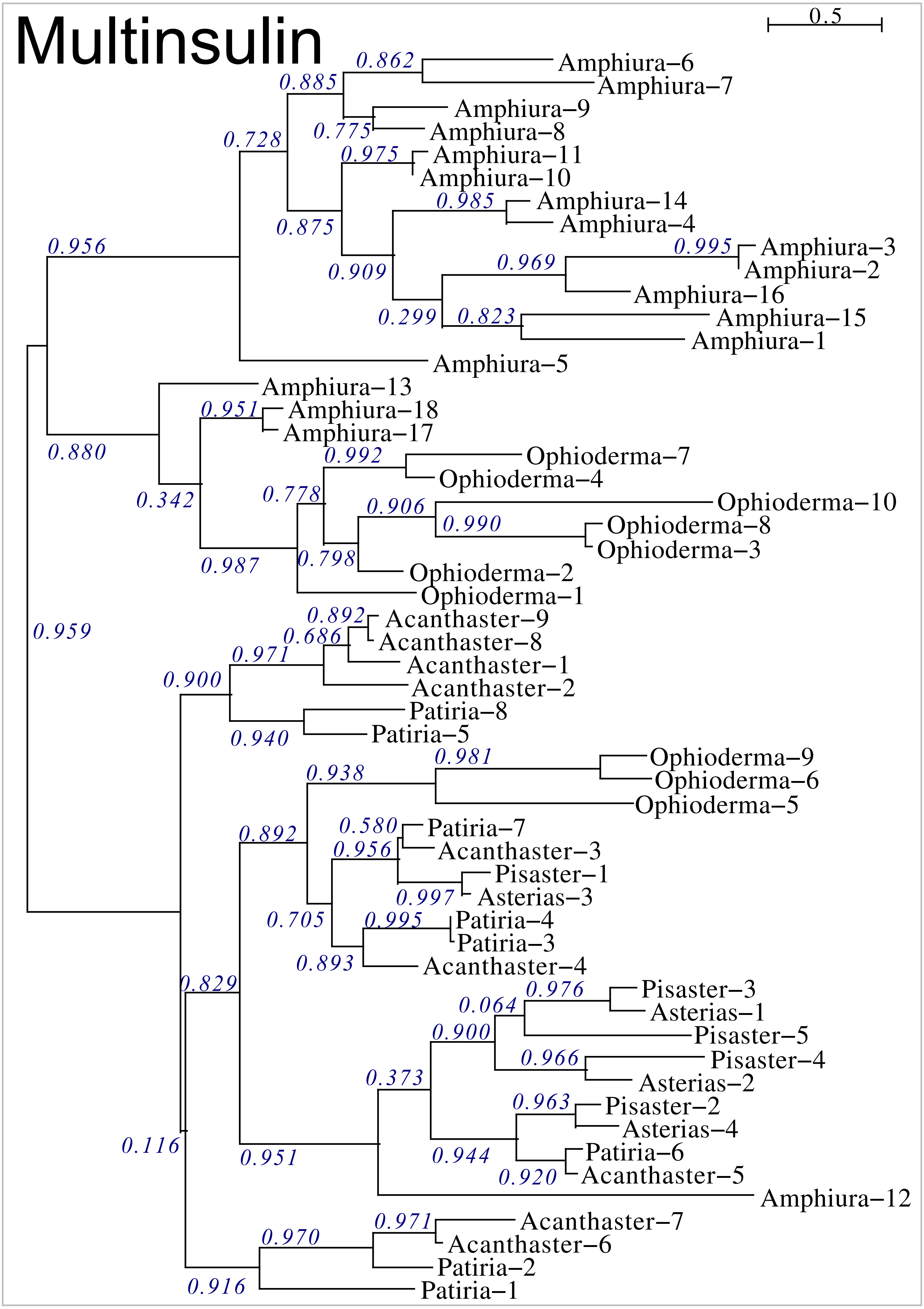

Figure S10. Sequence similarity tree of ambulacrarian multinsulins.

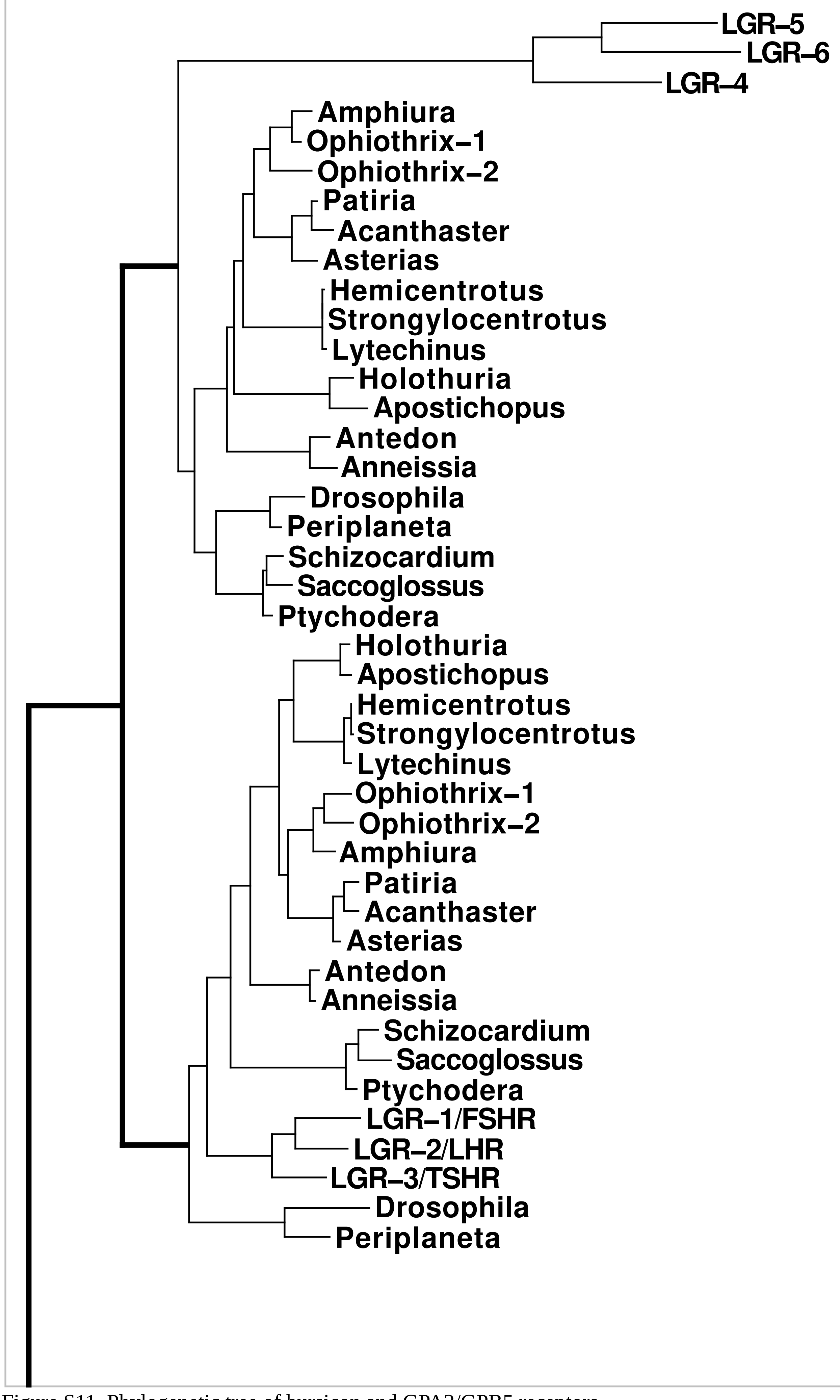

Figure S11. Phylogenetic tree of bursicon and GPA2/GPB5 receptors.

```
Ptychodera           ----------------------------------------------------------
Periplaneta          ----------------------------------------------------------
Anneissia            ----------------------------------------------------------
Apostichopus         ----------------------------------------------------------
Strongylocentrotus   ----------------------------------------------------------
Patiria              MEMKEPAEDGNKTKIRAGSDGTPRPRNILTSRSSRASRSSRGDGFRGSVWSAASSMLH

Ptychodera           ---------------MFIVITLLATLPG-------------------------VTSNE
Periplaneta          ----MQCRRSVACGLAVLAVVLA---------------------------LAALTFLL
Anneissia            -------------------------------------------MCSSKLAGLICIFVL
Apostichopus         ----------------------------------MLTRTGKWILLSVTM----IALTS
Strongylocentrotus   -------------MKMEL-ILRVVTFTSA-L--AAMSRTGSCQ--------------W
Patiria              RLRTRQCKVLVLCLVLLLILVLAVTFIGVSLTGNDVAQPGNNEFIPATVKTEYEELIG

Ptychodera           LPQSFSCPDGYFQCGNV-TECVHQKYFCDGKADCVDGFDEIPTKCRDNFGWANLISRI
Periplaneta          SQTHGACAEGTFPCGNS-SICLPQRFICNGHIDCPNGEDESKYTCTDVHGSNFNQDIV
Anneissia            LPFTYCCQSGTFPCGDDKEQCFPQEFQCNDVVDCTNAADEQ--DCEEKAGFGDDFDRR
Apostichopus         PCWAFECPKGEFVCGNL-TKCVKQKYQCNEIDDCGNNADEEEEVCRNFEGWVKSMDKV
Strongylocentrotus   ANDLIECDPGFFPCGNS-TECVAQQFQCDDIAHCQNAADES--GCAIADGWLAHFDYV
Patiria              FSSEYTCPKGEFRCGNL-THCVAQKYQCNGEDDCGNNADET--ECEHNEGWIKTFDEQ
                     LDLa

Ptychodera           TTFDKSNNDTLQPRNFIRRCELDDYPEACECT-FTSVSCANANLTGIPTNISGSVTHL
Periplaneta          IN----RNY----SQAFNDCQLESLPRGCLCRDKTWVFCRELGLTHVPQDISTNVTRL
Anneissia            HNTTIVLKYD-VGNYNTNVCLIPVYPPVCKCGNKTEVDCSATGMTTIPQAISRNISRL
Apostichopus         F----DEKD-----NIERECELTNYPPVCDCVNGEKLICSNKGLTEVPSVPPNT----
Strongylocentrotus   HPVSVGETK------ALNECALAQYPDGCVCSKLTQLDCSEFNLNEVPPNLPENITRL
Patiria              NPVPVDLDW-----QINQECGLYGFPDVCVCFEETELKCIQVNLTQVPQSVSANLTRL

Ptychodera           YVSGNKIKRLEPGIFKEYPNLLTLSLFDNAVEKLEEDTFQGLPRLEKLYLNDNKIKEI
Periplaneta          ILSNN-SITLSQDSFKKYHNLELIHLGWNQISDIPNGMFKGQRTLRRLFLSKNSLTEL
Anneissia            TLLGNDIQTILNNSFVNFEQLHKLTLAQNGLVRMEINAFNGLTELKQLYLSENNIKEI
Apostichopus         ---------------------SHLILMNNQIHDIPLTAFEGLTNLKVLYLSENPISTL
Strongylocentrotus   YLKSNNIVNIGSELLANYSSLVYLNLMGNRIHEIAENSFRNLRNLKRLFLSENNISRI
Patiria              NLNGN-MVHLEDGAFERYTKLGSLNLMQNGIRELPRDVFRGLTDLDKLYLSTNRISTI
                     LRR

Ptychodera           EDGAFRSLQKLAWLMLNNNHLKSLGEGMFEGLESVYYLNLKDNEVTEADFGTLTFTHM
Periplaneta          RPGVLQDLPHLSWLVLQDNCISDIDLDTFQYL-NLTWLDMTRNNLT--LKGK-IFPDL
Anneissia            PSGVFLPLKRLEWLVLDYNELTTLLGNPFKGLLSLYWLSVRHNKINDANIRT-VLENL
Apostichopus         RAGVFRDLINLEWLFLVNNTLRSLDDDAFIGLRKVYWLRLNSNLLEDLSDGT-PFRHM
Strongylocentrotus   QTGAFNGLSGLHWLFLNDNKMRTLEPGIFLGLEGLYWLDMRNNEIRDLQEGE-ALKDL
Patiria              TPGTFRFLQNLTWLFLNENEIETLDEDVFEGLHKVYWLMLQKNKIGDLNHGV-AFRDL
                     LRR

Ptychodera           PSLEWLNLGDNDIKHLHTGMF--PNNSALKVLFLGSNLIQSIHEDTLRGLDTLEDLDL
Periplaneta          PELKQLILEDNTIETIGEFTL--TNLKALQALDLTKNRIRYIHPKAFANLWNLEELEL
Anneissia            PSLAWLELDHNKITRIEAEDF--ARDTPMSTLSLKNNEVSYVHPLAFTSLINLLDLDL
Apostichopus         QELKWCDLGSNKITKITSGTF--QGETGLQTLDLSNNKIHTISKGAFLGQDNLTVLNL
Strongylocentrotus   KSLVWLDFEGNDIRSINARSF--AGNTSLTVLILRNNSIESIERDAFTSLGTLVELDL
Patiria              PKLMWLDMSNNPLHHLSADNFSASGNPPLSILMLSNCNISTIQKDTFQAFRELTALHV
                     LRR

Ptychodera           SDNLLSTIPAKTFHELISLAKLNLCNNRKLRRLPLELFSNLHQLRTLNLTGVKITNID
Periplaneta          SGNQLVSLSSSVFYNLTFLMKLSLSNN-PFRLLPTQLFRRLENLRSLGLVDTDIQNID
Anneissia            CGNKISELSPMQFKNLTKLDRLALGFN-LFDSLPLNIFDDLVNLQSLRLEGITINNID
Apostichopus         TLNNLSSVPKGLSSELPMLTTLSLSFN-PIQ-LDELSFSGFHNLKEIDLVGVDIPNIN
Strongylocentrotus   SHNQISHLPEGIFDGLHTLEILRLRNN-PLFSLPVDVFRGMKKLVDLDLAEIIIGNIN
Patiria              SGNKIGHFPSGLFRNMANLTVLTIANN-PATSLPEDLFDDLVLLDDLNLGGLVINNIS
                     LRR

Ptychodera           TKMFNSMQKLEHLYFAKFHYCSYASHVRDCSPKSDGISSLENLLANKVLRCFIWIVAA
Periplaneta          VELFAHCPKLEFLYFSKFYYCTYAPAVRICRPNTDGVSSLKHLLVKPALRVTVWVVAA
Anneissia            QRMFEKMEKLSIVYFSSFQYCSYAPHVRNCNPKTDGISSIYNLLESTVLRVSVWTIAT
Apostichopus         NAMFEQINSLTILHFERFEYCKFAHHVRNCNPKTDGISSTENLLEGAILRISVWTIAL
Strongylocentrotus   VDMFQGLTRLEKVAFDKFEYCRYAPHVRTCTPRSDGISSFENLLKNVVLRVFVWTIGI
Patiria              ARMFKGMTNLQHIEFFKFEYCRYAAHVRTCKPRSDGISSFENLLKDGILRVSVWTIAL
                     Hinge region
```

Figure S12. Sequence alignment of various gonadulin receptors.

```
Ptychodera          TTLVCNLAVILGRMCIMKIENKVHSLLIKNLCGADLLMGIYLVIIGSHDLMYRNHYYI
Periplaneta         VTCAGNALVLWGRF-SSRDENRVHSLVIRNLAVSDLLMGVYLLVIGTQDCQFRDRYHK
Anneissia           MTFIGNFGVFISRL-LMKAEHRVYSLFIMNLCAADFLMSIYLFTIAAQDYRFRDVYNQ
Apostichopus        LTTCGNLNVFISRI-AIHAENKVHSIVVSNLCAADFMMGLYLFAIGIQDARFRNNYID
Strongylocentrotus  ITSVGNLGVLVSRA-FMKAENKVHTVVVINLCTADFLMGIYLLIIGTHDVKYRDNYNQ
Patiria             LCFLGNIGVLISRY-LMKAENRIHSLVVVNLCTADFCMSIYLFVIGYHDAKFRNQFNT
                      TM1                                   TM2

Ptychodera          YSHKWMTSWTCSLCGFLAVFSSETTLLVLTVISVQRFLCIAFQTRMIH-FKYSYGVIS
Periplaneta         EAHSWMTSWKCTVVGMVAMTSSEVSVLILVFLSMERFLLIAVPFGGHQNMSMRTTCLS
Anneissia           NARQWVESTGCKMSGFLAMVSSEVSVLLLALISVERFLCIVFPYEFHR-LKVKEAAVI
Apostichopus        YALDWSESWGCHFVGFLALLSTEASVLTLTFISLERFFIVAFPYRFHR-LNIKEVVSV
Strongylocentrotus  HALEWTNGITCKVSGLLAMISSEVSVLTLMFISLERYFIIVYPYSFQR-IKTRRAIMV
Patiria             HALEWMQGSACKFAGFMAMFSSEVSVFMLTFISLERFICIVYPYRLHR-LTSKEALVI
                                      TM3

Ptychodera          IAAAWITAAALAAFPLTM-SSFNHFYGKNGACFPLLDPNDPFEAGWQYSVAMFLGLNA
Periplaneta         LAVIWFIGVCLAAVPVMYWINSTRFYGTNGMCFPL-HIDDPFLLGWEYSAFIFLGVNF
Anneissia           ISSIWAVGILIAWIPLLAEGYFGNFYGTNGVCFPL-HIHGPRIKGWEYSVAIFLGMNL
Apostichopus        LILIWIVAFVLAASPLIFTEYFKNFYGANGVCFPL-HIHDPRLEGWEYSIFIFLGINT
Strongylocentrotus  LAFIWFFGTLLAVVPIIPVDYFGNFYGSNGVCFPL-HLHEPRMRGWEYSVVVFLGINS
Patiria             MTIIWFLGALVAWIPLISVGYFVDFYGSNGVCFPL-HIHDPWLQGWEYSAFIFLGLNA
                         TM4

Ptychodera          VCFCLIVCSYVGMFYSIRETRNAAFKDINLRRRGDMNFAKRFVFIVLSNALCLVPIML
Periplaneta         SSLLLIAVLYAWMFVSIWRTRHATP----ISSVGDFEFVIRFFFIVLTDAGCWAPIFA
Anneissia           LALILIIVSYIGMFISIRTTRKAAV---QIGYKGDMTYARRFFFIVLTDSLCWIPIII
Apostichopus        FSFMVIIFSYCGMFQSIQKTRKRTT---T-LLPGDINYAKRFFFIVLTDCICWLPLAV
Strongylocentrotus  TAFVAIALSYTGMFLSIRKTRRAAA---QCGMKGDMAYAKRFFFVILTDSLCWLPIAV
Patiria             TCFTAIAISYTGMFISIQQTRKAAT---HLGRRGDMNYAKRFLFVVMTDALCWLPIAI
                     TM5                                                 TM6

Ptychodera          ITAMVFTDIGVPGQVYAWTTVFVVPINSASDPLLYTLTTVPCKQGCLGMLDKKLKRQK
Periplaneta         LKLVALGRVHIPADLYAWVVVFILPVNSAVNPLLYTFTTPKYRGQLCQLG----CPPQ
Anneissia           LKVISLCNIPIGDNLYAWIVIFVLPLNSALNPILYTLSTTTFSQWFLKLL----KIHK
Apostichopus        VKVLAVADMPVSESIYAWVVIFILPINSALNPILYTLSTGPFRRVLLQKI----KNRG
Strongylocentrotus  LKVMSLCNFIISETLYGWIIVFVLPINSALNPILYTLSTTSFAMWFMRQL----KRGK
Patiria             LKILSLCSYEIPATLYGWIVIFVLPINSALNPILYTLSTTSFTQWFQKHL----KRRK
                                    TM7

Ptychodera          ESGTRKMLVSSTTGSRSSRDSSGTTRLTSSGS-----SGSPSQDT-------------
Periplaneta         TLATKRHSSADTELSRSSTNRNAVDRRADN----GLYAMLPARDPAHT----------
Anneissia           QVS---HNYVTYSPQRTARRKSGIAEENGSTDGMMSYANRPSLQTNCS----------
Apostichopus        KHT---ETGAIPKNERFSNGTN---DTAGETLRLQ--SNLTM-----MGGNKHEANK-
Strongylocentrotus  FKT---SGCYIGSNVDMSELRG---RIEESGA-FTSYTNMPKRASFFLPTRKHHVSSK
Patiria             GKS---SNGSLGSKNEFSTTRG---RIGSTTDQPTEYSSIPKRSYDSNNS--------

Ptychodera          ---KPCIEM----DTAQPLVQN--------SSVGQIQSGQS----------------C
Periplaneta         ----------------------------------------------------------
Anneissia           ----DLLSTAISEDAAEDVRNNTQYKAQHSD--------------NEQPAST-YFVTC
Apostichopus        -----T----MAENTC------------------------------------------
Strongylocentrotus  VEAIPAVSSSVPSRSATTTAMTNNIRPLHTINEGEIVDNHSQSQGNHQLRRTPIVRLC
Patiria             ---SPVLEEE----QCSTV---------------------------------------

Ptychodera          A---
Periplaneta         ----
Anneissia           V---
Apostichopus        ----
Strongylocentrotus  DLME
Patiria             ----
```

Figure S12-continued.

```
Melibe              MEESITPREGSGSEAEESGSTTTATAKLWQHTRARTHYLVFLGVMICVALIVVLVCVT
Drosophila          MSIAI-----------------------------MPHLPITFTLAILLAIASNEGAQG
Periplaneta         ----------------------------------------------------------
Anneissia           MEY-S-----------------------------DKCRPFTKVLHCLLVLQLFFSFHL
Lytechinus          ---------------------------------------MNLALLMVVVTSFFDTSLA
Strongylocentrotus  ---------------------------------------MNWAVLMVVVSSFFGTPQV
Apostichopus        ----M-----------------------------KTMALICHKILQVLIAALFLGV--
Holothuria          ---------------------------------------MMTNILVFLIISMVTRD--
Acanthaster         ------------------------------------MRKLHNALRMLFVLHLCA----
Patiria             ------------------------------------MHKLRNALRMLLILHVCA----
Saccoglossus        ----------------------------------MNLIGDVRKLKYLNTALR-A----
Ptychodera          ---------------------------------------MKKSLRFLLILSL-V----
Shizocardium        ----------------------------------------------------------

Melibe              ST-------------------------TEK-----------------AEALTNALGAS
Drosophila          VESATRTAIEAIRTGIGTKPETEIADATEAEAPVREVISLLGIIDGAESDILVPDADD
Periplaneta         -------------------------------------MFLLQVLF-AVITVTETVGTF
Anneissia           VL--TC-------------------RVI-------------INTCPDVQEVTSKSPHG
Lytechinus          SH-NTK-------------------DDHLDDA--------P-VIRHRRQAPMSTDPHA
Strongylocentrotus  SN-ATK-------------------GDDLDDA--------PSLIRYRRQSPMTSDPHA
Apostichopus        ---TFA-------------------GKEVDQS--------VELHRVRRQ--SSSDVHG
Holothuria          ---AVA-------------------TQGDLDV--------YGARRVRRQ--SNSDNHG
Acanthaster         ----SR-------------------RAEQDGA---------IIRHIQR----RAVDAHK
Patiria             ----AR-------------------RVKQDSA----------LRHLQR----RTVDAHK
Saccoglossus        ----VH-------------------GYSQRQR--------MGRVHQR----IV--RGT
Ptychodera          ----SS-------------------SVSKRGR--------LGHSPHR----RLPRSFF
Shizocardium        ----------------------------------------------------------

Melibe              RCPSNTFRCPSDGRCINRTLVCDGRKQCTDGADEGTVCD--KDKMD-------LWSDM
Drosophila          KCPGGYFHCNTTAQCVPQRANCDGSVDCDDASDEVN---CVNEVD--AKYWDHLYRKQ
Periplaneta         LCKEGQFKCNKSRLCVPQDRNCDGIPDCPDKSDEWD---CSDKLG--SQFWDHLYRKN
Anneissia           DCPGFRFPCQNSSQCVPGNFICNGISDCDNGSDELP-EECATNNPLYTNIWTHIYGSE
Lytechinus          HCGE-WFPCINSTQCIPQEAICDGNIDCDNESDESD-EECIDDKN--RRVYTFMDQGK
Strongylocentrotus  HCGE-WFPCINSTQCVPQEAICNGIFDCDNKMDESD-EECIDDIN--RRIYTFKDQGK
Apostichopus        HCQVTEFPCGNSTECVLQSKHCDSVVDCANGADEDE-EDCDDMDN--TNNFDFLFGKK
Holothuria          HCGL-DFVCGDSMQCVTQSQQCDGVADCDNGADEEE-EECTDEDD--YRHFDEIIGRK
Acanthaster         HCGK-DFPCLNSTQCVPQDSICDGTPDCDNGSDEWEVEECRDWNL--AKMWDNFFGTK
Patiria             HCGV-DFPCLNSTQCVPQDAICDGTPDCDNGSDEWEMEECRDLNL--AKMWDDFFGTK
Saccoglossus        CSGI-QVKCTNSTQCIFRDQLCDDHVDCDNGSDEWS-SECTDEDD--NTNWDILIGQK
Ptychodera          CLDN-SFKCLNSTDCIFREYLCDGIEDCDNGSDEWD-EECTDYND--GENWDIKIGDK
Shizocardium        ----------------------------------------------------------
                                        LDLa

Melibe              FNKRLDADR-------------------------------------------------
Drosophila          PFGRHDNLR-------------------------------------------------
Periplaneta         PSAELDEED-------------------------------------------------
Anneissia           RRGGDGDSSDED----------------------------------------------
Lytechinus          TDDSSEEDD-------------------------------------------------
Strongylocentrotus  QDDSSEEED-------------------------------------------------
Apostichopus        ED-S--EER-------------------------------------------------
Holothuria          EE-L--DEL-------------------------------------------------
Acanthaster         NDDSGEEEEDDLDQGLRPQ---QTVFDAHGHCGEEFPCMNSTQCVPREAICNGKPDCD
Patiria             D-ESGEEEEDDVNQGLSPDPLPQRSFDAHGHCGEDFPCINSTQCVPQEAICNGEPDCD
Saccoglossus        SDSEEEED--------------------------------------------------
Ptychodera          DDESSEEEGEDD----------------------------------------------
Shizocardium        ----------------------------------------------------------
                                                                   LDLa

Melibe              --------------------------------EKEAKNCVWDKIPSRCVCR-------
Drosophila          -----------------------------------IGECLWPNENFSCPC--------
Periplaneta         -----------------------------------L-VCELMINSSECAC--------
Anneissia           --------------------------------FNPDNVCENGTYPPNCTCYLKCISRH
Lytechinus          ---------------------------------FIPPACDIGTYPEECRCVLLYEDPM
Strongylocentrotus  ---------------------------------FIPPACDAGTYPDVCRCVLLHEDPM
Apostichopus        ---------------------------------PPPVTCTDTIFPDECECFVVFDEES
Holothuria          ---------------------------------PPREPCNGTVHPEECKCFVVAEE--
Acanthaster         NGSDEEEIV-CRNKKM----------RDNIEDIRQEIPCEEGTFPESCDCFVEIEQTR
Patiria             NGSDEWELEECRDWNLAKMWDDFFGDKAEDEDVRREIPCEKGTFPESCDCFVEIEETR
Saccoglossus        -------------------------------NESETIECDNGSYPEECNCTHFILREN
Ptychodera          -------------------------------LGGSRIPCAKDTYPNVCNCTYIEFENG
Shizocardium        ----------------------------------------------------------
```

Figure S13. Sequence alignment of dilp7 receptors.

```
Melibe              ----------------------------------------------EKGKLYC---RD
Drosophila          ----------------------------------------------RGDEILC---RF
Periplaneta         ----------------------------------------------VERSVLC---NN
Anneissia           GGN-------------------A-------------------DDGECESEVFCYTENT
Lytechinus          AHF------DYYES----MLQDA--YP-----SVFAEPPIGATSEPLGLEIAC---TS
Strongylocentrotus  VNF------DYYES-----LQDV--YP-----SAFADPLFGESGEPMGLEIAC---TS
Apostichopus        ---------------------------------------GLENDEPIGLQLDC---QL
Holothuria          -------------------------------------------GEIIGLELLC---PQ
Acanthaster         TAELPSLASPYNTTGDASTYRDA--YDIDSNSTDGVRYGSEVRGVVIGIRLDC---RA
Patiria             -KDLPSLASPFNATGDASMGAGVDDVGTETNVTDGIPYGLGVQGVVVGVRLDC---SA
Saccoglossus        GFM----------TSE----------------N--------QFYTIIDTRLDC---SH
Ptychodera          HNA----------TSR----------------SDE----HPNDNSPYAIHIDC---SD
Shizocardium        ----------------------------------------------------------

Melibe              QDLSAIAHPLPQYT-SVLELSGN-ITSISRLSIGRLPSLHTIIVIHSRVKEIGALTFD
Drosophila          QQLTDIPERLPQHDLATLDLTGNNFETIHETFFSELPDVDSLVLKFCSIREIASHAFD
Periplaneta         MGLTLPPPLLPARGIELLDLTGSSFPVLNATIMDTLPDLEILVLSHAGIKELQQEAFS
Anneissia           TGFTTVPRDLPTNT-VLLDLSDNYISVVRKGDLDHLPYLRTLYFSRNRNLRFESDALT
Lytechinus          KRLERVPEGLPPGT-LYMDLSDNRITHLHGDSFQNLTRLRELVITHNQMQSMDEDVFY
Strongylocentrotus  KQLDRVPEALPADT-LYLDLSDNRITHLHDDSFKNLTMLRELVITHNQMQTMDEDVFY
Apostichopus        SELESVPDNLPRDT-VYLNLAGNSLSNITVETFKTLTKLRRLDLQSNRIGSLHPDSFR
Holothuria          SGLTSVPRGLPRET-IYLNLAANEITTIDKDSFDEVTSLKRLYLQSNKISILHPQSLN
Acanthaster         KQLTNVPRNLPNNT-ISLDLSDNKITHLTKKDLTNLPQLRQLLLSRNKLKRMEEGAFK
Patiria             RQLTEFPRNLPNKT-ISLDLSDNKITSLTKKDMEHLTHLRQLLLSRNKLQNVEEGTFE
Saccoglossus        RNLTSIPGGIPPNI-TKLNLRYNSIQEVKAGDLYGLYDLTELLLSRNIINDIAERALD
Ptychodera          RNLTAVPQNIPDNT-TEIDLSQNSIETIRKADFAGLFMVKNLLLSKNSIREIEEGSFD
Shizocardium        -----------------------MIQEIGSEDLGGLVMLRELLLKKNNLTKLNSSSLS
                    ========================= LRR ============================

Melibe              EC--------------------------------------------------------
Drosophila          RLAD------------------------------------------------------
Periplaneta         KA--------------------------------------------------------
Anneissia           ----------------------------------------------------------
Lytechinus          DTR------------------------------------------------SLEMLDL
Strongylocentrotus  DTT------------------------------------------------SLEMLDL
Apostichopus        QLR------------------------------------------------SLELLDL
Holothuria          KLL------------------------------------------------KLEILDF
Acanthaster         PLT-------------------------------------------------------
Patiria             SLT-------------------------------------------------------
Saccoglossus        HLANLMVLDLEANKLKTLPESVFSFLNNLVDLDLSKNDLGTLPIYIFKNLNKLQKMDL
Ptychodera          DLRDLLILSLDRNKLTSLPASVFQTVTNLRELSLELNDISNIPDTVFQNLGHLEELDL
Shizocardium        DLVNLEKLAIESNKLTTIPVDIFSKMKNVQILTLAKNDLQHLPVGVFASLRRLEELDL
                    ========================= LRR ============================

Melibe              -----------------------------------------PSLANVHFTFNKLSRFS
Drosophila          -----------------------------------------NPLRTLYMDDNKLPHLP
Periplaneta         -----------------------------------------PSLHTLYLDNNQLRTLP
Anneissia           ---------------------------------------NNLTISELNLVGCQMGTIP
Lytechinus          SDNRIERITSAHFDSLRRLSLLRLSRNH-FRCEDGDCFRNLVSLRELELVACGITELT
Strongylocentrotus  SDNLIEIITAAHFDNLQKLTLLRLSRNH-FRCEGGDCFRNLVSLRELELVACGITELT
Apostichopus        SNNRISSLSKEYFLFNGILKELLLKHNPIQRLEDAELFINLQMLETLDFFGAQISELR
Holothuria          TNNKISWLGSQHLQQLFSLKELTLKHNPIRRLNDSDMFRHMYSLQTLNFLGAQISELR
Acanthaster         ------------------------------------------DLETLRMIACDLEDVQ
Patiria             ------------------------------------------DLQTLRMIACDLEDVP
Saccoglossus        SGNEIRLI-QSELFLGQGSLDVLHLGWNRPLEISEHAFSALRNLTRMRLLDNGLSHLR
Ptychodera          SGNEIRLL-GKNLLRNQVTLKKLQINWNRPMEIEFGAFDNTVNLNTLRLIDNGISHIR
Shizocardium        SGNELRRL-DKDLFENQSGLKTLQLNWNKPLELEFGVFDRLKDLQILGLLDTGLSHIR
                    ========================= LRR ============================
```

Figure S13-continued.

```
Melibe                SSVFAKRNSIIYLNLMGNAIKELTSGDFRRMKSLKKLDLRDNKITSIDEGVFEDTPHL
Drosophila            EHFFPEGNQLSILILARNHLHHLKRSDFLNLQKLQELDLRGNRIGNFEAEVFARLPNL
Periplaneta           EPALQPGNILDTLILRNNELSKLPKNAFLNLQYLRELDLRHNHLADVDGDVFQPLVRL
Anneissia             DGLFSGMSHLMTLHLQYNNLVTINKDTLKGLDWLMELNLNHNEISDLNPMAFNHTPNL
Lytechinus            PDMFRNLHSLSVLQLSYNAITEIMPSHMNGLEGLQDLYLSSNHISHIENGSFDGMDRL
Strongylocentrotus    PDLFSNLHSLSVLQLSYNAITEIIPSHMNGLERLQDLYLSSNHISNIEHGTFDGMDNL
Apostichopus          TGVFSPLENLRKLVLAHNEIEALTQGNMEGLRNLLELDLQNNEVSFIEDGTFKEMPML
Holothuria            TGIFEPLTKLTRLVLSHNQIEALTQGNMAGLSSLQTLDVQSNDISFIEDGAFIELESL
Acanthaster           QRMFAAQKRLVTLDLRYNKLTRLVKNSLFGLRNVKYFDIRGNQLSEIETGVFEDTPKL
Patiria               PRMFATQNRLVTLDLSYNILTRLVRGSLFGLHNLEYLDIRGNRLAEIETGVFKDSSRI
Saccoglossus          SGMFRNLNRLKELDLERNQILSIKKYDLQGLTDLSHLKLKNNTIRRIEDGAFDHTPSI
Ptychodera            SGIFRNLNKLTKLDLERNFILSIKKYDLQGLHNLSSLQLRNNSLTRIEAGAFDHTVKL
Shizocardium          SGIFCNLGKLTKLDLERNKLLSIKKYDLEGLDNLKDLLLTNNTIQRIESGAFDHVGKL
                                          LRR

Melibe                ISLDMRGNLLTELVPGMF-DGLQDLRKLYLGNNAIAEIAPGAFASLSKLRAINLARNR
Drosophila            EVLYLNENHLKRLDPDRFPRTLLNLHTLSLAYNQIEDIAANTF-PFPRLRYLFLAGNR
Periplaneta           EILYLNENRLDSVPL---SLPLPSLQLLSLTHNHIAWIEPGAFSQLPRLKHLYLSWNQ
Anneissia             VILNIQYNKLQVLSPPLL-EPLTQLKTLGMSFNQISEIKNGFFESQTHLQDLLLMGNK
Lytechinus            LKILLHDNKLKNISVGVF-PFMPELVILKLDENEIDMIEPGSFSLVMNLQHFTLMENK
Strongylocentrotus    LKLLLHDNKLTNISVGIF-PVMPELQILKLDENEIAIIEPGSFALIENVQHFTLMKNR
Apostichopus          NELRLHGNKIHEVKQGMF-EDLANLTILLLDQNLIHTIEPGSFSKLFKLQHITMSNNM
Holothuria            EELRLHGNKLTKVKRGMF-TNLAQLRTLLLDQNEIESIEPGTFAELTNLQHFTISDNL
Acanthaster           YFMLVSQNKLSSIPANLL-RPLRELRTLDVHRNDISVIETGAFSTNTKLIELNLRDNK
Patiria               YFMHISQNRLSSIPAELF-RPLRDLRSLNIHRNDISSIECGSFSTNTKLRDLNLGDNK
Saccoglossus          SELWLSMNDLTEVKRSVF-LPLKNLKTLDLSFNKIEEFEDGALSNSVILAILLLAGNR
Ptychodera            KQLRLSLNTLPAIKKDVF-EPLQELKILDLSHNKIEDIESGSLSNSTRLSHLLLASNK
Shizocardium          ETIGLSFNDLVEIKKDVF-SPLKEIRSLDFSFNKIQRIETGAFANSRLMTHLFISDNN
                                          LRR

Melibe                IRSLRDGGLDGLGNLTTLDLHYNGMHTIESHAFKSLVSARSVDLRSNHLISIGNGTFS
Drosophila            LSHIRDETFCNLSNLQGLHLNENRIEGFDLEAFACLKNLSSLLLTGNRFQTLDSRVLK
Periplaneta           LWVLRNDTFDNLPELLSLSLNGNKIRLLEVNTFKEVPLLTSLYLQDNQLKLLDSRVLR
Anneissia             LTVIKVGMFQNLYGLLSMTLRENNIREVQANSLDHIESLQSLSLAKNPFESLPDHVFC
Lytechinus            LSRIELGMFDGLENVTDMSLRQNEIKAIEENAFDNLIRLDHY-LRENKLTEVPRELFT
Strongylocentrotus    LSRIEVGMFDGLGNVTDMSLLENEIKVIEENAFDNLIKLESLDLQKNKLTEVPRELFT
Apostichopus          LTHLTVGMFDGLSQVSSLSLYANKITEMENGTFEPLIKVSSLNLKDNLFRVVPRGIFD
Holothuria            LTHLPVGMFEGLSRVVSLGLFDNHIETMENGTFAPLLNVESMNLERNPFRIVPRGVFD
Acanthaster           LTEIRRGIFHSLTSTITLSLSNNSIRHLEQDAFAGMNNLQTLKLTKNPFTSLPVGIFD
Patiria               LTEIKRGLFHNLTSVITLSLRNNSIRHFEEDAFAGMNNLQTLKLNQNPFTSVPIGIFD
Saccoglossus          LPFVRTGMFANLTKVSNLPLFDNEIAWFDADSLQDMESLRTLDLQHNPVQRLPMVLFD
Ptychodera            LSILRNGMFTSLSSVTNLDLLDNEIRSLEPGCFDDMLSLYTLDLRENPFTLLPRGLFD
Shizocardium          LHDIRIGMFSGLATVTNMELNNNDIEVVEAGAFDDMTSLYTLDFQKNPFTRLPRGIFD
                                          LRR

Melibe                YMH-QLHYIYFDKFHMCLYAQHSQQCLPLGDGISSKENLLESIVLRICVWVVALLSLG
Drosophila            NLT-SLDYIYFSWFHLCSAAMNVRVCDPHGDGISSKLHLLDNQILRGSVWVMASIAVV
Periplaneta           HLQ-NLEHIYFDEFKLCSAALHVRVCEPKGDGISSLQHLLDSIVLRASVWVMAVLACA
Anneissia             AMTNTLDTIEFDSFSFCGFAPHARHCYPSTDGLSSRDHMLKSVILRISVWIVGSIALF
Lytechinus            PLE-NLNNIYFDYFFMCGYAPNVRVCLPKGDGISNVDDLLGNWLLRGAVWLVALLGSF
Strongylocentrotus    RLK-NLNNIYFDFFFMCGYAPNVRVCLPKGDGISNVDDLLGNWLLRGAVWLVALLGCF
Apostichopus          VMP-MLGHIYFDHFFLCGFAPHVKDCDPKSDGLSTSEDLLGSIVLRVAVWLVSILASF
Holothuria            VMP-KLSHIYFDHFILCGFAPHVKDCNPKGDGVSNSENLLGSIVLRVSVWMVSILATF
Acanthaster           QLI-SLKAIYFDHFSLCGYAPHVRLCMPKSDGISTAENLLGNILLRFAVWFVALLASL
Patiria               KLR-SLKAIYFDHFSLCGYAPHVRLCMPKSDGISTAENLLANNLLRFAVWFVALLASL
Saccoglossus          DLP-SLEWVHFDHFWMCGYAPTVRHCTPRGDGISSVENLLDNVVLRVMVWVVAMLAFV
Ptychodera            NLL-SFEWIYFDHFSLCGYAPTVRHCSPRGDGISSIENLLDNMVLRIMVWVVATLAFI
Shizocardium          KLGESLQWIYFDHFSMCGYAPTIRHCEPRGNGISSIENLLDNMVLRIMVWVVALLAFI
                                                                       TM1

Melibe                GNMVVFLGRF-LLREDNQIHSFFIKNLSMADLLMGLYLLIIAIHDIKFRGQYILHDES
Drosophila            GNLLVLLGRYFYKSRSNVEHSLYLRHLAASDFLMGIYLTLIACADISFRGEYIKYEET
Periplaneta           GNTVVLLGRLVARE-PNPVHSLYIKTLALADLLMGIYLMIIASADWHYRDVYVRHDFQ
Anneissia             GNLFVLTTRLFVNE-TKRVHAFLIMNLAFADLLMGIYLYLIAIHGALFRDNYILYDLV
Lytechinus            GNLLVILARCFVSE-DNKVHSFFIMNLAVADFLMGLYLLIIGLHDVMFRGEYIFKDLN
Strongylocentrotus    GNLMVIFARCFVSE-DNKVHSFFIMNLAVADFLMGLYLLIIGLHDVMFRGEYIFKDLD
Apostichopus          GNLIVLFARCVSKE-DNKVHSFFIINLAISDFLMGAYLFIVAVHDVMYRGVYILHDVD
Holothuria            GNLVVLFARCLSKD-DNKVHSFFIINLAVSDFLMGAYLFIVAIHDVIYRGSYITYDLD
Acanthaster           GNAFVLLARCFVNE-DKKTHSFFIMNLAVADLLMGLYLLIIGIHDVIFRGSYILHDLT
Patiria               GNAFVLLARCFVKE-DKKTHSFFIMNLAVADLLMGLYLLIIGIHDVIFRGVYILHDLA
Saccoglossus          GNLFVLVARCILKE-DNRIHSFFIMNLSLADLLMGLYLFIIALHDVMYRGEYIKFDLE
Ptychodera            GNIFVLIVRCVLKE-DNRVHSFFIVNLSFADLLMGLYLFIIAVHDVIYRGEYIKFDLS
Shizocardium          GNIFVLIARCILKE-DNRVHAFFIMNLSFADLLMGIYLFVIAIHDVIYRGEYIKYDLS
                                           TM2
```

Figure S13-continued.

```
Melibe               WRNSWECDFAGVLSTLSTEMSVLTLSVITLDRYISIMYPLSLRRRGLRVAYFVMALTW
Drosophila           WRHSGVCAFAGFLSTFSCQSSTLLLTLVTWDRLMSVTRPLKPRDTEKVRIVLRLLLLW
Periplaneta          WRHSPVCNACGFLSTLSCEASVLILTLITRDRLVSVTRPLDRRQPSLRHATLLVAVLW
Anneissia            WRSSDICTIAGCLSILSSMMSVFTLSVITLDRYVSIVHPFSYESKSLFRAAILMGVLW
Lytechinus           WRSGWVCRMCGLLSLLSSEMSVLTLTVITSDRFISIVHPFKFRQRHLAHAVILMVAFW
Strongylocentrotus   WRSGWVCKMCGLLSLLSSEMSVLTLTVITSDRFISIVHPFKFRQRHLAHAVILMVGFW
Apostichopus         WRSSWVCQLCGAFSLLSSEMSVFTLTIITADRYFCIVHPFRFRSRNLIPAAILMGCLW
Holothuria           WRSGWVCQMCGAFSLLSSEMSVFTLTVITADRYFCIVHPFRFRNRQILPAAILMGVLW
Acanthaster          WRNSSVCKLSGFLSLLSSEVSIMTLTVITLDRFLSIVHPFRFKNRSLVHARLLMVFLW
Patiria              WRTSSVCKLSGFLSLLSSEVSIMTLTVITMDRFLSIVHPFRFKNRSLVHARLLMVLLW
Saccoglossus         WRLGWVCQMCGFLSLVSSEASVLTLAIITMDRFICIVYPFKFKNRNMKIAVFAMLLIW
Ptychodera           WRTSWICKLCGFLSFVSSEASVLILTVITLDRLFSIVYPFKFKNRTIKLAFLIMATMW
Shizocardium         WRLSWVCQLCGFLSLVSSEASVLTLTIITLDRFICIVYPFKFKNRNLKLAFWVMLMVW
```

TM3

```
Melibe               TVCIM-LVSLPVMG--LEYFGDSFYRDNAVCTPLHLHHPRAKGWEYSSFLFLGINFCS
Drosophila           GISFGL-AAAPLLP--NPYFGSHFYGNNGVCLSLHIHDPYAKGWEYSALLFILVNTLS
Periplaneta          LVAAVV-AALPLCAETVEYFGEEFYGGNGVCLPLHVQDPFADGWEYSAAMFIALNTVA
Anneissia            LFGITL-VLIPIIM--KQTFGELFYGSNGVCLPLQIRHPWATGWVFTTTLFVGLNSVF
Lytechinus           LAAMLI-AILPVLH--RSYFGEFFYGGNGVCLPLQFDRPFDHGWEFTLVVFVLFNLIA
Strongylocentrotus   LAAMLI-AILPVLH--RNYFGEFFYGGNGVCLPLQFDRPFDHGWEFTLVVFVLFNLFA
Apostichopus         IIGITL-SILPLLH--RSYFGDFFYGANGVCLPLQFDRPFDDGWLFSLFVFVVANLLS
Holothuria           LLGITL-AMLPLVY--KTYFGDFFYGANGVCLPLQFDRPFDDGWVFSLFVFVIANLLS
Acanthaster          LLGIAL-ATIPLLH--TAYFGEFYYGGNGVCLPLQIDQPFADGWEFSLVIFVVFNLVA
Patiria              LLGVAL-ATIPLLH--TEYFGEFYYGGNGVCLPLQIDQPFANGWQFSLVIFVVFNLVA
Saccoglossus         ISCVII-ATLPLID-AITYFGDFFYGGNGVCLPLHIDDPKADGWEYSLVIFVLVNFMA
Ptychodera           SICIFL-AVLPLID-HLEYFSVFFYGGNGVCLPLHIDEPMTNGWEYSLFIFTFINLVA
Shizocardium         LACVFL-AILPLIH-VITYFGAFFYGGNGVCLPLHIDEPKANGWEYSLFIFVLVNFVA
```

TM4 TM

```
Melibe               FAFIAYAYVAMFYSIHMSALPLRTSRDSKERCLVQRFFFIVLTDFVCWVPIIIIKILA
Drosophila           LIFILFSYIRMLQAIRDSGGGMRSTHSGRENVVATRFAIIVTTDCACWLPIIVVKLAA
Periplaneta          LLFISFAYWRMLRVVRSSGLSLRSTQERQDKAVAQRFVIIVATDCLCWIPIIGVKLAA
Anneissia            FLFVVYAYIAMFVMIRRSD-DVRSTKKSQDSAILVRFTLLVFTDMLCWLPICVIKVLA
Lytechinus           FIFILYAYARSKAIPRMNKMGKRSTKESQDWNLLKRFTIIVATDFICWMPIILVKIAS
Strongylocentrotus   FLFILYAYAQMFATVRKSSLAMRSTKESQDWNLLKRFTIIVATDFICWMPIILVKIAS
Apostichopus         FTFIVWAYIRMFFTIRRSNLAARSTKVSQDYALLKRFTVIVATDFLCWMPIIIVKFVT
Holothuria           FLFIVWAYIRMFTTIHRSNLAARSTKVSQDYALLKRFTVIVATDFLCWMPIIIVKFVT
Acanthaster          FTFISYAYLMMFMTIRRSNLAMRSTKKNQDWALVKRFTLIVATDFVCWMPIIIVKFVA
Patiria              FAFISYAYLMMFVTIRRSNLAMRSTKKNQDWALVKRFTLIVATDFVCWMPIIIVKFVA
Saccoglossus         FLFIVFAYMAMFTSIKKSRSNIRSTKENQDLLLVKRFTLIVATDFICWMPIIIIKFAA
Ptychodera           FLFIAFAYAAMFNAIRQSRTNIRSTTENQDYMLAKRFTLIVATDFVCWMPIIILKLVA
Shizocardium         FVFISFAYVAMFSSIRKSRTNIRSTKENQDLVLVKRFTLIVATDFICWMPIIIVKFAA
```

M5 TM6

```
Melibe               LSGVAITVDLYAWVIVFILPVNSALNPLLYTLTTQYFKKKLFSKFSAVVFRPVLRSD-
Drosophila           LSGCEISPDLYAWLAVLVLPVNSALNPVLYTLTTAAFKQQLRRYCHTLPSCSLVNNET
Periplaneta          LAGARVDQQLCAWLAVLILPVNSALNPVLYTLTTSFFKSQMSRLLHAWKSRDTGPSHD
Anneissia            ISGYPVSGDVYSWLAVFVLPVNAALNPILYTITSKLFRQQLR----IILHKCRRATGY
Lytechinus           YAGIVIPQSVHAWFAIFVLPVNSALNPILYTVTTQFFRQKFLRPIRRICGRQKQGKAY
Strongylocentrotus   YSGIAIPQSVHAWFAIFVLPVNSALNPILYTVTTQFFRQRFLRPIRRLCGRRKQGKTY
Apostichopus         YGGVAIPPAAHAWFAIFILPVNSALNPILYTMTTRQFKRMIFHPSRTFMGKQKSTAGH
Holothuria           YGGVIIPPTAHAWFAIFILPVNSALNPILYTMTTRQFKRMLFHPSRALMGMSKSAGGN
Acanthaster          LGGVSVSQSVYAWFAIFVLPINSALNPILYTMTTVLFRQKILAPLGIVKAKRK--KGY
Patiria              LGGVSVSNSVYAWFAIFVLPINSALNPILYTMTTVLFKQKVLAPLGIVRAKRK--KGY
Saccoglossus         LAGAQISGGVYAWVAVFILPVNSALNPILYTMTTKMFKQKFMKLLGISNKKKN--AG-
Ptychodera           LAGVKVNGDIYAWMAVFILPVNSALNPILYTMTTKLFKQKMLRALGVHLRD----KA-
Shizocardium         FAGAKINGDVYAWIAIFILPVNSALNPILYTLTTRMFKQKMLRAFGVATK-----KE-
```

TM7

Figure S13-continued.

```
Melibe              ----------KDS----N----------------------QSRSSMKNNTLYSRHMHD
Drosophila          RSQ----------TQTAYESGLSVSLAHLGGGVGGGSGRK--RMSHRQMSYL------
Periplaneta         SAG------------SLSNIQLSTRKRHLGSQPPTRSSTL-----RSHSSYKSIRGT-
Anneissia           RRG---RRDDDSDSFKRSGTRLSLI--PVRSKASLKSKSS--IDKTKSNS--------
Lytechinus          QSG---SYDDSITGTKITATKLSII--SHNGKPRAGSVNG--RLNSTKGSHGHHRP--
Strongylocentrotus  QSG---SYDDSITGTKISATKLSVI--SHNGKPRAGSVNG--RLISTKGSHVHHRP--
Apostichopus        EMSQSMSRTRDREIGHTATSRLSVI--SHSNTMKNGT---------------------
Holothuria          DMSQSVSRSRDREMGNTATSRLSVI--SHSHQMKNGGGTA--TPG-------------
Acanthaster         ITG--VSVDETSTMSKNSGTRLSII--SNKS--RGGSLNG--R-----------FNSQ
Patiria             LTG--ISVDDTSTMSKNSGTRLSII--STKS--RGGSVNG--R-----------FSSH
Saccoglossus        --------DESYSASKTTGTRLSSV--ASSR--NRISLNG--NIKIYTYSIPFICSTI
Ptychodera          --------DESLTASKTTGMRVSTF--GQR---SRSTTST--NGKARNGSFHDNVQDN
Shizocardium        --------DDSFTASKTSGTRLSST--GSR---TRISISA--K--NSNGSIGTRATSL

Melibe              LEMDLLRRYGNASRSGT----QTTTTTLSTSR---IPHNGKATSVRPRAQSDNALNIR
Drosophila          ----------------------------------------------------------
Periplaneta         ---------------AV-----------------------------------------
Anneissia           ----------------------------------------------------------
Lytechinus          ---SEVQPWLSPDSSGRPSHLKRPSLRMEDMSCRKE-----------------PEVVV
Strongylocentrotus  ---SEVQPWLTPDSSGHPSHPKRPSVRVEDTSCREE-----------------PEVVV
Apostichopus        -----------------PTTPRNG--HMENLSHED-----------------------
Holothuria          ---------------GTPAAERNA--HMENMSNDD-----------------------
Acanthaster         KKLKNLSSLDSTDESVTCSAAQTT---SLKIKKHRA-----------------A-TAD
Patiria             KKLKNFDSLDSSDESVICTATKTT---T----KKRA-----------------V-TVE
Saccoglossus        FRLKTILFFIIKKSDTPAS--STT---LRDIKLAAA-----------------PKSLR
Ptychodera          -QFDRDVAFDNQGADMS----------GEPIALSVQ-----------------AEHLR
Shizocardium        RTTSREQTYPVKDCNYV----------ELTTIYTPP-----------------PE---

Melibe              PNVGNA---SQPISNI-----------------
Drosophila          ---------------------------------
Periplaneta         ---------------------------------
Anneissia           ---------------------------------
Lytechinus          VHSNSQN--RIECSDVAEPLIET----------
Strongylocentrotus  VHSNSQN--RIESSDVVEPLIET----------
Apostichopus        ---NTNN--EDRDQNMDSPE-------------
Holothuria          ---NKKE--RNEE--MAAL--------------
Acanthaster         YHELPTSDP-DCAPSGVNDDME-----------
Patiria             YHQLPVSDS-DERPLASTVT-------------
Saccoglossus        RTLIEQGDGSSEVRSAAAIEIQRAWRGYWARWS
Ptychodera          GTE------------------------------
Shizocardium        ---------------------------------
```

Figure S13-continued.

```
Apostichopus        ----MMRILQSVVFLALLLLM------------------VVNISQSSPPDC-EETDIF
Anneissia           -----MEH---RLVPMLLAIAWISLC------------VVSKSELHGSVSC-KSTDVK
Strongylocentrotus  ---MMMG-RQAFIFLVLGIA------------------LVDIVAGVNLQQCDVEVEFE
Patiria             -----MR-LTSILLVVFLISLFLA-------------SDARLRNKDETTSCENEADFE
Ptychodera          -----MGRYLGSCHRFLLYVIWFS---------------VTVAETQHQVVCDIDTELE
Periplaneta         MLFQLLPNKASLVFVICLQIVYICSSTNEPVLGSGVMHGAVPGGSVVMPCNSSEAVFH
Lymnaea             -----MA-TMSGTTIVCL--IYLT-----TMLGNSQGVNLKIESPSPPTLCSVEGTFH

Apostichopus        CDN---KCLNVTMQCNNVYDCVGGEDESFLICGCRHDEFTCSD-GCIPLHERCDLNCS
Anneissia           CGT--GECVAANSSCNSISDCPDGSDESPAYCGCRHDEFQCYN-SCISLFGRCDGTCD
Strongylocentrotus  CEP--GVCIPRNLTCNGIFDCELGQDEEVAFCGCAKDEFPCSH-SCIPIELRCDTNCD
Patiria             CEP--GVCIDASLACNSAYDCESMADETVMLCGCPSGEFRCLN-SCIPIEKRCDTNCD
Ptychodera          CSP--GVCIDLQYRCDGIPDCENGHDESTLVCGCPSHEFQCNETTCIDLIRRCDWSMD
Periplaneta         CDA--VVCVPKSSLCNGIPDCQDGRDESVAQCGCLPNEFRCSN-SCVDLVRRCDRQPD
Lymnaea             CDDGMLQCVLMGSKCDGVSDCENGMDESVETCGCLQSEFQCNHTTCIDKILRCDRNDD
                                              LDLa

Apostichopus        CSS-CEDEDECEGYSCPPTHN-KCK-NHYCIPQINFCDFIDDCGDNSDEENCNHPPCS
Anneissia           CDF-CEDERDCDLHQCPSTHM-KCATSHICIHLSLQCNFVDDCGDGSDE-MCEHRECY
Strongylocentrotus  CEVDCADMDGCEAFPCLEGYE-KCTKNHYCIAKHLWCNFVDDCGDNSDEDLCYHPDCW
Patiria             CQDDCADEDHCDSFQCLPGFQ-KCE-NHFCMPTYAWCDFVDDCGDMSDEALCERLDCW
Ptychodera          CPD-GSDEDNCETFQCLHTHS-KCQNTHKCIPRDKTCDFIDDCGDKSDEEDCYRRPCY
Periplaneta         CAD-GEDEQDCEAYVCPT-THFKC-NNHFCIPGDSVCNFVDDCGDGSDENKCSHRKCW
Lymnaea             CSN-GLDERECDIYICPLGTHVKC-DNHFCVPRDKQCDFLDDCGDNSDEKICERRECV
                                              LDLa

Apostichopus        SMFLFECENHQCISNAYACDGIEDCFDGSDER--HCEEEN-FHICEDGNKIHEGRKCN
Anneissia           T-HEFQCANGECLKPGYRCNGEVNCRGGDTSDEDNCIENYDYIMCR-SGRIFRNLFCD
Strongylocentrotus  K-GEFQCSNKQCINTWFVCDGSQDCIDGSDE--ARCGEEH-FVLCENGKKVYEHEWCD
Patiria             Y-GQFQCINQQCINSFFLCDGIQDCTDGTDEMESTCSPGN-FADCGDGTRVRAQQFCN
Ptychodera          AYGEFRCENEQCISSYIVCDGINDCLDGSDEV--QCQQED-FVTCSDGTQIHRHQWCD
Periplaneta         N-LEFKCNNGECIRPGFVCDGTQDCKDGSDET--DCGPDQ-FALCGDGSRVHRYYWCD
Lymnaea             A-TEFKCNNSQCVAFGNLCDGLVDCVDGSDEDQVACDSDK-YFQCAEGSLIKKEFVCD
                                              LDLa

Apostichopus        MVRDCPSGS-DEHGCEC--QVYQTQCPAEKSCFDNDIHCNSFCECAD----------C
Anneissia           FFIDCELTEEDEKQNCSCDSADGFMCS-TGVCILRSKLCDGQCDCRDL---------C
Strongylocentrotus  RLVDCPDNEADETNCVC--TADEYRCP-NGKCLRPSVRCNGVCDCLS----------C
Patiria             GDAKCQLNKAVEINCDC--SDDEFRCG-NGKCLPPAKQCDRLCDCSN----------C
Ptychodera          GIPDCRESHDDEVNCYC--AEDDFRCG-NGRCISRAYLCDLSCDCAGT---------C
Periplaneta         GWPDCPDNHADELKCQNCSGVDEFLCP-NGRCIRRANVCDSQCDCVPPPGSAAVNVTC
Lymnaea             GWVDCKLTFADELNCKLCD-EDDFRCS-DTRCIQKSNVCDGYCDCKT----------C
                                              LDLa

Apostichopus        SDETNCDEI---------CDV-SNGIYCPWMNYYIGSYRLSGCVKTERFCDGTPDCLG
Anneissia           EDENNCGDT--------VCSA-GSDYFCA---------HSFRCLNSEFVCNGYNDCEN
Strongylocentrotus  DDEKECADEDM-------CNYETNGILCQ-----IANNKPSRCVRKEYICDGFGDCPN
Patiria             EDEEGCDNKDD-------CDTDT-HIFCP-----PVDGKPPVCLQKQFICDGRRDCPI
Ptychodera          EDEKNCSSG--------LCTL-ENHFWCD---------EFSRCLHKDTICDGKFDCFD
Periplaneta         DDEVDCDLYYTNINGIHLCTQ-TETLNCIEP---NQNRDKDRCIHREFICDRTNDCQN
Lymnaea             DDEEVCANN---TYG---CPM-DTKYMC------RSIYGEPRCIDKDNVCNMINDCRD
                                              LDLa

Apostichopus        Y---QDEMFCSVPLHVP------------VRQC---PSTRDPRYIPES-EFCDN--SS
Anneissia           KDDE----LCESRYKTD----VGYECKLNSDMCQL---DNNAFCYPTE-IFCER--RP
Strongylocentrotus  K--AD-EFNCQL--PGNTTGHCDSEGNPPYMMCDIKDGADDPRCLPTLKVQCDRYRRP
Patiria             F--GWDEDDCQTLPDGESVASCNVPG---TVSC----GAGDPRCPPSKYVKCDK--RF
Ptychodera          ---WSDEYGCGDDVNCS---THEYENQMEFFRCG-GVESDDGRCIPER-AVCDY--QR
Periplaneta         GNYLSDEFGCPDDLDVI----------GESIVC------SDNRSLPKA-LECDF--KW
Lymnaea             GNVGTDEYYCSNDSECK---N--FQAAMGFFYC------PEERCLAKH-LYCDL--HP
                                              LDLa

Apostichopus        YCFQGSEETNCTHGERVCSVGQFQCADS--SCVEESLRCNGFWDCKDFSDEIDCPNYI
Anneissia           QCQGGSNGSHCL-VEALCNSNEFKCTNG--MCIPDYLRCDNIADCLDHSDELNCESHP
Strongylocentrotus  HCMYGFDEETCNATRPCDPEVEYECPYG--RCIDLTSRCDAQLDCFDFSDEANCESFE
Patiria             DCLTGVDEQNCTV-MPCPPGQHQCRRGG--YCVKEELWCDGYVDCFDGSDEDNCELFE
Ptychodera          HCILGTDESDERCGHPACMEDEFQCNNGHVKCIPIVQRCDTNPDCLDGSDERNCEDYL
Periplaneta         DCLEGNDEHG-CV-PDACAEGEYTCKNG--QCVNSTQRCDLQFDCWDKSDELNCVNEK
Lymnaea             DCINGEDEQS-CLAPPKCSQDEFQCHHG--KCIPISKRCDSVHDCVDWSDEMNCENHQ
                                              LDLa
```

Figure S14. Sequence alignment GRL101 receptors.

```
Apostichopus        CPNENMFKCRGTGQCLASHQRCNGEVECTYINGT-KEDSDEENCV--LRDCEENEFRC
Anneissia           CEE-GMWKCR-EGHCIEMHMRCDRYVDCANFGQY-RDASDEENCD--FESCKQNEFQC
Strongylocentrotus  CLP-GTWKCH-SGQCIPEKQKCDYTPDCFYVNGT-VDSSDEDKCDYKIFGCREGEFRC
Patiria             CPP-GLRKCA-AGQCISQRLFCDYHLDCFYYKNSTRDVSDEAHCT--RRQCREDEFQC
Ptychodera          CDE-GQLQCNESGQCIHRDMLCNFIKNC-------ADNSDEENCGHVIRNCTEDEFQC
Periplaneta         CPS-GKKKCVHSGQCVSAAFWCDFIVDC-------PDGSDETDCGEKPPLCGPNQFRC
Lymnaea             CAA-NMKSCL-SGHCIEEHKWCNFHREC-------PDGSDEKDCDPR-PVCEANQFRC
                    LDLa

Apostichopus        HSGHCIENWRRCYKAPDEHLYGCKDRSHLWDCGQWECADESQFKCPRDHCISTMSRCN
Anneissia           NNGRCIDESKKCFRDY--SSEECRDNSHLRECEDFECPA-GYYKCQKSHCIPDFRVCD
Strongylocentrotus  HSGQCIPQDEVCFFDST-MKKGCKDRSHLNDCANRTCRE-NEFKCRNAHCVNMSDVCN
Patiria             RSGQCIPASRRCYRDQHDHVQGCADLSHLRNCSDFVCPA-GAFKCARSHCINGTMVCD
Ptychodera          RNGQCIDLSRRCYFDELDDVYGCKDKSHLIGCNDFECSA-DTLKCRNGHCVSQSLVCN
Periplaneta         NNSQCIDASLRCFN-SGNPRYGCADGSHLIGCKNWTCPS-NAFKCRNGPCLNSSMVCD
Lymnaea             KNGQCIDPLQVCV--KGDKYDGCADQSHLINCSQHICLE-GQFRCRKSFCINQTKVCD
                    LDLa

Apostichopus        GLLDCVNNE-DEA-----NCLMYCHPA----KYPGCFCKGKIFNCSYTGLTQVNIDHF
Anneissia           VHVDCLLFYDDEV-----NCYGKCGTDFEFYSIPDCICNDKTVDCNDLKYGTLDFKEL
Strongylocentrotus  GAVDCLPYWTDED-----FCPHECGP-----A-LMCICNHTQMSCINAGLESFHNFYV
Patiria             KNVDCLESWDDEAPRHGDTCIQECAS-------SGCGCSQFDMTCTNIGLNESSFSLN
Ptychodera          GNIDCGLIWTDEE-----GCSHICSN-----L--VCMCQDDEMNCEDKNLHEIPYF-I
Periplaneta         HNIDCPGTWEDED-----GCLFACSN-----VEPRCECRDIHVNCTNLGLVRVPPD-I
Lymnaea             GTVDCLQGMWDEN-----NCRYWCPH-----GQAICQCEGVTMDCTGQKLKEMPVQQM
                    LDLa

Apostichopus        A--DDEVTWFYFNHNQLTV--SALTFGNSTVQNIVHLDLSHNNLQDLPAGLFSMMIGL
Anneissia           ----KDMNIMKFSGT----ELVLESSTFSNVTAITFLDISRNNLMDIGDGYFKDMFFL
Strongylocentrotus  ---EEQISEINLSGNRIQ----ISNEVLSGLPGLLRLDLSNNSLNDIGNDTFKGLANL
Patiria             AKDTRLIIHFSLPGNNLHH-LLQDPTALEGLTRTILLDLSDNGVTFINKAALLNVPNL
Ptychodera          ---EPGYIKLKLGHNYLGEDPNLSNYTFAKLGRLTYLGLEDNNITDLKSFTFSNLWRL
Periplaneta         ---EEEITWFHMANNLINS--TFSEDTFEQLERLLYLDLRNNSISSIPPLAFRNLWRL
Lymnaea             ---EEDLSKLMIGDNLLNL--TSTTFSATYYDKVTYLDLSRNHLTEIPIYSFQNMWKL
                    LRR

Apostichopus        HYLNLGYNGINKLEAQVFSGLTNLKQLVLTGNVIQIIEEGSFLGLTSLKVLDLSHQRL
Anneissia           QTLILSENKITTLKKGAFQGLSNLRYLNLTKNIIENIEADAFDGLDSLTTLDLSYQNI
Strongylocentrotus  RYLNLENNNLRVIRKQTFNGLEGLQTLRLGGNNIHAIEPHAFEGLRNITTLDLSNQNL
Patiria             QTLILADNKITKLGNQTFSSLQHLRTLILRGNAMQTVAGQAFLGLGELTTLDLSQQKI
Ptychodera          LTLNLEDNFLTTLRRDTFDGLGNVRSLYLQGNSIGEIEAHAFKGLSALPTLDLHGQRL
Periplaneta         LTLNLQYNCIRILSNSSFYGLTNLSNLHLQGNGIQVIEAMAFYGLSSLILLDLSWQRI
Lymnaea             THLNLADNNITSLKNGSLLGLSNLKQLHINGNKIETIEEDTFSSMIHLTVLDLSNQRL
                    LRR

Apostichopus        IHIGGNPFVGLYHLTLLNISNNNLQTVEQGIFLKLDNVHTIDMSHNQINFIHENTFNG
Anneissia           QRLPINAFYGLRSLKYLTLRGNKLIFIVGGTFNGLPNLRSLDISENTINKIDGRAFNG
Strongylocentrotus  TEIPNAAFVGLYRLHFLDLSANHIVMVPDGAFYGLYQLKKLDISENAIEVVSRR-TFN
Patiria             QSINKGAFDGLNKLAFLNLSHNRMTDIPRGTFFGLNELKELDISNNKLSQIHAEAFYG
Ptychodera          QWLHINAFAGLSRLLSLNLSHNQIQYIDNGVFNGLSKLLVLDISHNVVIEIDSKVFYG
Periplaneta         HNISQGAFVGLRSLVGLDLSHNEITYLVDGSFNGMPHLLSLDLSSNTIKVISSNVFRS
Lymnaea             THVYKNMFKGLKQITVLNISRNQINSIDNGAFNNLANVRLIDLSGNVIKDIGQKVFMG
                    LRR

Apostichopus        LTNLEKIISDELRFCCVAKH---VKECLPEADEFSSCEDLMRNTFLRFNLWVVGLVAT
Anneissia           LPSLDYLYTDEFRFCCMSEG---VPNCYPEPDAFSSCDDLMSNYVLRVSIWVLGVTAF
Strongylocentrotus  LRLLEELITDEYRFCCMARH---VSKCLPLPDEFSSCEDLMSNPILRMCIWIIGMIAL
Patiria             LPKLRVLHTDEFRFCCMVREMVSVETCTPPPDEFSSCEDLMANVFLRGSIWVIGIIAS
Ptychodera          LMELTELYTDEYRFCCMAKF---VQKCHPPPDQFSSCQDLMSNVVLRGGIWLLGVIAS
Periplaneta         AASLTKLVTDEFRFCCLARH---VKHCLPPPDEFSSCEDLMSNLVLRICVWVLGVLAT
Lymnaea             LPRLVELKTDSYRFCCLAPE---GVKCSPKQDEFSSCEDLMSNHVLRVSIWVLGVIAL
                                Hinge region                       TM1

Apostichopus        FCNAIVIVTRSGKRRDSRVQSFLITNLAIGDMCMGIYLLIIAIADLNYRGRYAAYESL
Anneissia           LGNLLVIIWRVTHNRENKVHSFLITNLAAGDFCMGVYLLIIAIVDLTYRGEYIIHDME
Strongylocentrotus  IGNLVVIMWRVNSKRDNKVHSFLITNLAVGDMLMGIYLLIIAGVDAYYRGDYIVHDKT
Patiria             IGNLAVIMMRMNNKRDNRVHSFLITNLAIGDFCMGVYLLIIASVDAFYRGYYIIYDAQ
Ptychodera          VGNLVVVCFRLRDRRDNKVHSFLITNLAVGDLCMGVYLIIIAVADTYYRGNYIIHDKV
Periplaneta         FGNILVIGWRMRFKHTNQVHSFLITNLAIGDMFMGSYLLIIAGVDARYRGVYFIHDSD
Lymnaea             VGNFVVIFWRVRDFRGGKVHSFLITNLAIGDFLMGVYLLIIATADTYYRGVYISHDEN
                                      TM2
```

Figure S14-continued.

```
Apostichopus        WKRSLFCKFAGFLSTFSSELSVLSLTFITLHRVSSIVFPFRTKDIGFSRIVWIMTSTW
Anneissia           WRSSSWCRFAGFLSTFSSELSVFSLTIITLQRLTSILFPFRSKNMELGWAMKMMALTW
Strongylocentrotus  WRNSGLCKFAGFLSTFSSELSVFSLTIITLHRLSSIVFPFRIKDMEFTRAVWVMCVSW
Patiria             WRDSALCRFAGFLSTFSSELSVFSLTIITLHRLLSILFPFRIKDMEYSGAVRVMVVTW
Ptychodera          WRQSQFCKIAGFLSTFSSELSVFTLTVITVDRLVCIVFPFRFNRLEFKGAARLMAALW
Periplaneta         WRSSELCHLAGFISTFSSELSVYTLTVITLDRFLVIIFPFRVRRLEMPKTRLLMALGW
Lymnaea             WKQSGLCQFAGFVSTFSSELSVLTLSTITLDRLICILFPLRRTRLGLRQAIIVMSCIW
                                         TM3

Apostichopus        AIVFFLAGIPLLGFSYFDNFYGRSGVCLALHITPDKPNGWEYAVFVFLALNFFSFTFI
Anneissia           LLAIFLAILPLCRVNYFGNFYGRSGVCLALHITGEKTSGWEYAVFIFLAINLLSFIII
Strongylocentrotus  GLVAFLAALPLCGVAYFGNFYGRSGVCLALHITPDKPSGWEYSVFIFLGLNFVSFITI
Patiria             LMVIFLSAFPLFGLSYFGNFYGRSGVCLALHITPDLPKGWEYSVFIFLALNFISFLTI
Ptychodera          LIVFFISAVPLFGLSYFTDYYGRSGVCLALHITNERPDGWEYSVFVFLIINLVNFLII
Periplaneta         LGAAFLSGLPLLQIDYFRNFYGRSGVCLALHITPEKPSGWEYSVFVFLFLNLISFSII
Lymnaea             VLVFLLAVLPLLGFSYFENFYGRSGVCLALHVTPDRRPGWEYSVGVFILLNLLSFVLI
                      TM4                                              TM5

Apostichopus        AVAYMVMFYVARRTQQAAKRSLRGNKGSDAMARKMTLIVFTDFCCWMPIIILGVASLC
Anneissia           ILSYVIMFVVARRTQRAV---R-PQNRGDSMAMRMTVIVLTDFCCWMPIIFLGIASLL
Strongylocentrotus  MVSYGVMFNVARKTQKAAMRSL-KSKGSDSMARRMSVIVFTDFCCWVPIILLGLASLS
Patiria             AVSYSVMFVVARRTQKAVNRSR-DTNTGDAMARRMTLIVMTDFVCWVPIILLGVASLG
Ptychodera          LLSYIAMFIVASKTQQAV-RNR-DLKTESAMAKRITVIVMSDFFCWVPIILLGLASLG
Periplaneta         ALGYLWMYVVARTTQQAV-KKE-QRPSDNAMARRMTLIVATDAACWMPIILLGVLSLG
Lymnaea             ASSYLWMFSVAKKTRSAV-RTA-ESKNDNAMARRMTLIVMTDFCCWVPIIVLGFVSLA
                                                                 TM6

Apostichopus        GANIHPPVFAWVAVFVLPLNSAVNPLLYTLWTAPYARKIINSARSTFGRS--TATTEY
Anneissia           GASVSPKVYAWIAVFVLPLNAAINPLLYTLWTSPYVRRFLKKARPSMRSLST-YYTDT
Strongylocentrotus  GAYVPTSVYAWVAVFVMPVNSAVNPILYTLGSAITVRRAIQRFSINPSTT---VTSEY
Patiria             GAKIPPQVYAWVAVFVLPVNSAINPMLYTLLTAPYVRRVMSRARTSLNLSLSTVTTDM
Ptychodera          GAVIPPQVYAWIAVFVLPLNSALNPVLYTLSTPTFVKRTKKMADSIGESFRSRWRTDG
Periplaneta         GITVPPQVFAWVAVFVLPLNAAVNPVLYTISTAPFLGPARRGLTTFKRSCKLSLTMDQ
Lymnaea             GARADDQVYAWIAVFVLPLNSATNPVIYTLSTAPFLGNVRKRANRFRKSFIHSFTGDT
                                         TM7

Apostichopus        KLSANLSTS-------EKVSSTANGRRAQRGL--------ASTSN---------SK--
Anneissia           RHGSVREH--------HRSGNN---FKMRISQ--RSK-------------KTSLKFIP
Strongylocentrotus  KTVMN----------NS-SDRKQGGYKGRP--M-RTMSNTANENCKPA----TTTTTK
Patiria             KQVNH----------GDRQGRMCNGRKGWQKGI-RLPKADANKNAIKLNTIISQSDTE
Ptychodera          RTNSFTVSESQA-----RSS----------------------FSYTGIDRLDRCASVA
Periplaneta         RRTYSSTLGSTFANNCSRCELDYNLYPRLEQTNGKLQEGAGGCHYDGVEDLDSEQDMK
Lymnaea             KHSYVDDGTT--H---SYCEKK-SPYRQLELK--------------------RLRSLN

Apostichopus        -LPPEKSTGSIRS---SDVINDPEEGIRLHDVSHDKGNVYVNPNNNDD------RLGN
Anneissia           SSPPKNNNSTTNKRTDAGVITNDEQYNKLLSDQ-RNKDSYE-----------------
Strongylocentrotus  PKPPPAKEETIKM---IDLGK------RRHDNGDDGEK------------RALTSAGN
Patiria             PDEPATAQTRSEE---LNLLQ------DHHSPTQGAES------------GKEAEAGM
Ptychodera          SSNSATVAIRLRS------FS------GGHNGGADLTGCYDKRTGSDPSTRERCDSGK
Periplaneta         NEAPLFS------------LT------KRHQPHRWRSGSYHATNNVVPPSSSVSTPG-
Lymnaea             SSPPMYY------------NT------ELHSDS-------------------------

Apostichopus        HGNKDAYEKML-----SDKSMDIDGGHSSEE--------------G------------
Anneissia           ----------------------------------------------------------
Strongylocentrotus  NNEK------------------------------------------------------
Patiria             HT--------------------------------------------------------
Ptychodera          SVTFASDSRTWLRRALQAPPKDIKIVEKLREVIIIEFKPEEQSVGAMIIGFLELFSEA
Periplaneta         ------ATILWQSSSCRHGSSSIDTAVSA---------------HGEIIPLRRLHSDS
Lymnaea             ----------------------------------------------------------

Apostichopus        ----------------------------------------------------------
Anneissia           ----------------------------------------------------------
Strongylocentrotus  --------------------TSNNV---------------------------------
Patiria             ----------------------GNV---------------------------------
Ptychodera          QEKHFTRLRDMLKKLSSNDTAHENIIRMLWSGSVSDLLSSQAITEQFPGEFKWCICVE
Periplaneta         -------------------RGNGNK---------------------------------
Lymnaea             ----------------------------------------------------------
```

Figure S14-continued.

```
Apostichopus        ----------------------------------------------------------
Anneissia           ----------------------------------------------------------
Strongylocentrotus  ----------------------------------------------------------
Patiria             ----------------------------------------------------------
Ptychodera          YIEGVTLKTFAKDKIDTRELSRIVIQIAKALDHLRKHKIVYNNLSTSSIIIQRVGQES
Periplaneta         ----------------------------------------------------------
Lymnaea             ----------------------------------------------------------

Apostichopus        ----------------------------------------------------------
Anneissia           ----------------------------------------------------------
Strongylocentrotus  ----------------------------------------------------------
Patiria             ----------------------------------------------------------
Ptychodera          SRKVRPILFDFCKAVDLSVALSTDSEDVDHMNDVMGIARMLNDLCTTVMVTTDSEDTD
Periplaneta         ----------------------------------------------------------
Lymnaea             ----------------------------------------------------------

Apostichopus        ----------------------------------------------------------
Anneissia           ----------------------------------------------------------
Strongylocentrotus  ----------------------------------------------------------
Patiria             ----------------------------------------------------------
Ptychodera          YERTPLFPMSQSVKRNGLRADEVLRMCITECATEPPSAFDILNQLTSLMSDPDIVYYV
Periplaneta         ----------------------------------------------------------
Lymnaea             ----------------------------------------------------------
```

Figure S14-continued.
